# Arbuscular Mycorrhizal Fungi Modulate Variety-Specific Phosphate Transporter Gene Expression in Aerobic Rice Under Phosphorus-Limited Soil Conditions

**DOI:** 10.3390/plants15111675

**Published:** 2026-05-29

**Authors:** Debasis Mitra, Periyasamy Panneerselvam, Parameswaran Chidambaranathan, Amaresh Kumar Nayak, Anjani Kumar, Upendra Kumar, Priyashree Parida, Abhishek Kumar Sahu, Annamalai Anandan, Pradeep Kumar Das Mohapatra

**Affiliations:** 1 ICAR—Central Rice Research Institute, Cuttack 753006, India; agriparames07@gmail.com (P.C.); aknayak20@yahoo.com (A.K.N.); anjaniias@gmail.com (A.K.); ukumarmb@gmail.com (U.K.); priyashriparida8@gmail.com (P.P.); edu.abhishek98@gmail.com (A.K.S.); anandanau@yahoo.com (A.A.); 2 Department of Microbiology, Raiganj University, Raiganj 733134, India; pkdmvu@gmail.com; 3 Department of Microbiology, Graphic Era (Deemed to be University), 566/6, Bell Road, Clement Town, Dehradun 248002, India

**Keywords:** aerobic rice, P-transporter genes, *OsPT*, P uptake, soil enzymes

## Abstract

Phosphorus (P) fixation in aerobic rice cultivation severely limits crop productivity. However, the mechanisms by which arbuscular mycorrhizal fungi (AMF) regulate phosphate transporter (*OsPT*) gene expression across genetically diverse varieties under variable soil P regimes remain poorly understood. A controlled pot experiment was conducted to examine six aerobic rice genotypes, CR Dhan 201, CR Dhan 204, CR Dhan 205, CR Dhan 207, IR 36 (P-susceptible), and Kasalath IC459373 (P-tolerant), under three soil P levels (low: 2.68 ppm, medium: 8.81 ppm, and high: 12.84 ppm) with and without AMF inoculation. AMF colonization was significantly higher (50.19–63.63%), and sporulation was greater (24.29–30.28 spores per 50 g soil) in CR Dhan 204, CR Dhan 205, and CR Dhan 207 under low and medium soil P. All AMF-inoculated varieties showed 33–55% improvement in root architecture and 14.87–50.22% higher P uptake compared to uninoculated controls under P-deficient conditions. Ten of the 13 phosphate transporter genes (*OsPT2*, *OsPT3*, *OsPT4*, *OsPT6*, *OsPT8*, *OsPT9*, *OsPT10*, *OsPT11*, *OsPT12*, and *OsPT13*) were upregulated in CR Dhan 207 under low soil P conditions with AMF, and the broadest gene activation profile was observed across all varieties. These findings establish that AMF-mediated regulation of the *OsPT* gene network is strongly variety-dependent and most pronounced under P-limited conditions, positioning CR Dhan 207 as a priority genotype for mycorrhiza-assisted phosphorus management in aerobic rice systems.

## 1. Introduction

Phosphorus (P) is an essential macronutrient governing critical plant processes including energy transfer, nucleic acid synthesis, and root development. In aerobic rice cultivation, P fixation is a primary constraint to productivity. Although P concentrations in many agricultural soils are adequate in total, the majority is rendered unavailable to plants through sorption by iron, aluminum, and calcium oxides in acidic soils, or by calcium and magnesium in alkaline conditions [1,2,3,4]. Continuous application of phosphate fertilizers has paradoxically exacerbated this challenge by promoting further fixation, with up to 80% of the applied P fertilizer may be lost to the soil matrix [5,6]. India’s upland soils, where aerobic rice cultivation is expanding, are especially prone to P deficiency, with widespread P-limited arable land reducing grain yields through stunted root systems, delayed maturity, and reduced tillering [3,7]. Arbuscular mycorrhizal fungi (AMF) are the most widespread symbiotic microorganisms in agroecosystems, colonizing over 80% of crop plant species and extending plant root access to soil P through an extensive extraradical hyphal network [8]. In rice, AMF symbiosis has been shown to enhance P uptake, improve root architecture, stimulate soil phosphatase activity, and increase microbial biomass carbon (MBC) [9,10,11]. However, the magnitude of these benefits is highly genotype-dependent and mediated by soil conditions, with mycorrhizal responsiveness varying substantially among rice varieties [12,13]. In aerobic rice specifically, where roots encounter oxidized soil conditions comparable to those of upland crops, the ecological and molecular basis of AMF-mediated P acquisition is less characterized than in flooded systems [14]. Phosphate transporters belonging to the Pht1 family play a crucial role in managing how plants take up and reallocate phosphorus (P). Foundational studies have mapped out the structural properties and functional roles of individual transporters within this family in rice (*Oryza sativa*). For instance, Paszkowski et al. [15] identified the highly divergent *OsPT11* transporter and demonstrated its specific activation during arbuscular mycorrhizal (AM) symbiosis. Building on this, Liu et al. [16] provided a critical framework showing how high-affinity transporters such as *OsPT6* and *OsPT9* are regulated during P deprivation and functional symbiosis. At the molecular level, rice acquires P from the soil as inorganic phosphate (Pi) through a family of 13 phosphate transporter genes (*OsPT1–OsPT13*) belonging to the *Pht1* gene family [15]. These transporters operate across a spectrum of Pi affinities and tissue types—from root epidermal high-affinity transporters activated under P*i* starvation to low-affinity transporters involved in long-distance translocation [16,17,18]. AMF symbiosis introduces an additional regulatory layer: genes such as *OsPT11* and *OsPT13* are specifically activated during arbuscular mycorrhizal colonization and are essential for symbiotic Pi transfer at the periarbuscular membrane [15,19,20]. Despite growing evidence for the functional importance of individual *OsPT* genes, a systematic comparison of AMF-mediated regulation of the full *OsPT1*–*OsPT13* gene family across genetically diverse aerobic rice varieties under a gradient of soil P availability has not been reported. In conventional flooded paddy ecosystems, the anaerobic reduction of iron oxides significantly enhances the solubility and availability of soil P [21]. Conversely, in water-limiting aerobic rice production systems, the soil matrix remains largely unsaturated and oxidized, which radically accelerates P fixation onto iron and aluminum oxides, profoundly restricting P mobility and chemical diffusion toward root surfaces [22]. Because of these distinct biochemical constraints, rice cultivars engineered for aerobic environments must deploy alternative strategies to optimize P acquisition compared to lowland cultivars. While traditional check cultivars like Kasalath are known to harbor specific tolerance loci (such as the *Pup1* QTL/PSTOL1 kinase) for enhanced P uptake [23,24,25], and others like IR36 exhibit high sensitivity to P deficiency, how modern aerobic-adapted cultivars respond dynamically to non-flooded, P-limited environments remains poorly mapped. Consequently, we hypothesized that modern aerobic rice varieties—specifically the CR Dhan series (e.g., CR Dhan 201, CR Dhan 204, CR Dhan 205, and CR Dhan 207), which were systematically bred for water-starved aerobic ecosystems—possess distinct, highly responsive regulatory networks governing both direct (plant-mediated) and symbiotic (arbuscular mycorrhizal fungi-mediated) P-transporter (*OsPT*) pathways compared to traditional checks (IR36 and Kasalath). To test this hypothesis, this study provides a comprehensive profile of the 13 *OsPT* gene family members across structurally diverse genotypes under variable P regimes and mycorrhizal inoculation conditions. So, the study examined the differential expression of all 13 *OsPT* genes, alongside P uptake, root architecture, plant biomass, AMF colonization, and key soil microbial parameters, in six rice genotypes, including four aerobic varieties (CR Dhan 201, CR Dhan 204, CR Dhan 205, and CR Dhan 207) and two check varieties (IR 36, P-susceptible; Kasalath IC459373, P-tolerant), subjected to three soil P levels with and without AMF inoculation under controlled net-house conditions. This study aimed to identify the most mycorrhiza-responsive aerobic rice variety and characterize the molecular basis of differential P acquisition, with direct implications for sustainable P management in aerobic rice systems.

## 2. Materials and Methods

### 2.1. Experimental Site and Experiment Description

A pot study was conducted under controlled net house conditions at the Microbiology Section, Crop Production Division, ICAR-CRRI, Cuttack, Odisha, India (20°25′ N, 85°55′ E; 24 m above mean sea level) during the 2021–22 Rabi season. The substrate consisted of a low-P soil sourced from Krishi Vigyan Kendra (KVK), Santhpur, ICAR-CRRI, Cuttack (20°27′45.08″ N, 85°52′58.76″ E) (Supplementary Figure S1), with an initial available P concentration of 2.68 ppm. This soil was amended with K_2_HPO_4_ to establish a gradient of P availability. The adjusted soils were stabilized at field-capacity moisture for a period of three weeks. Subsequent analysis confirmed three distinct P regimes and categorized low (P1:2.68 ppm), medium (P2:8.81 ppm), and high (P3:12.84 ppm) available P (Supplementary Figure S1). A pot experiment was initiated using 3 kg pots with six rice genotypes, including V1 (CR Dhan 201), V2 (CR Dhan 204), V3 (CR Dhan 205), V4 (CR Dhan 207), V5 (IR 36, P-susceptible), and V6 (Kasalath IC459373, P-tolerant). The experimental design was a randomized complete block design (RCBD) with three replications for each treatment. The treatments combined the three soil P levels (P1, P2, P3) with two arbuscular mycorrhizal fungal (AMF) inoculation treatments with 50 g of soil-based mixed AMF inoculum [*Funneliformis* sp. (CRRI-CPD-AMF1), *Glomus* sp. (CRRI-CPD-AMF3), *Rhizophagus* sp. (CRRI-CPD-AMF6), *Acaulospora* sp. (CRRI-CPD-AMF7), and *Claroideoglomus* sp (CRRI-CPD-AMF8) received from Microbiology, ICAR-CRRI, India] per pot (AM1) and a non-inoculated control (AM2). The AMF inoculum, obtained from the Microbiology Lab, Crop Production Division, ICAR-CRRI, had a spore density of approximately 135–140 spores per gram of substrate (Supplementary Figure S3).

### 2.2. AMF Colonization and Sporulation

The protocol established by Phillip and Hayman [26] was used to assess AMF colonization in the rice root systems. Fresh root specimens were carefully cleansed of adhering soil particles and subsequently subjected to a clearing process, involving immersion in a 10% potassium hydroxide (KOH) solution, followed by autoclaving at 121 °C for 15 min. After decanting the potassium hydroxide (KOH) solution, the treated root specimens were rinsed. This involved three successive washes with tap water, and the process was deemed complete upon the observation of achromatic runoff, confirming the elimination of leached chromophores. The samples were then acidified in a 2% hydrochloric acid (HCl) solution for 5 min. Without a subsequent water rinse, the HCl was decanted, and the roots were stained using a 0.05% trypan blue solution (HiMedia, Bangalore, India) prepared in lacto-glycerol (400 mL lactic acid, 400 mL glycerol, and 100 mL water), with a second autoclave cycle applied for 15 min at 121 °C to facilitate staining. Excess dye was removed by destaining the roots in a lacto-glycerol solution after autoclaving. For assessment, ten segments of stained roots were mounted on a glass slide and examined under a compound microscope RxLr-4 (Radical Scientific Equipments Pvt. Ltd., Ambala, India). The extent of root colonization was measured as a percentage, according to the procedure outlined by McGonigle et al. [27].

### 2.3. Estimation of P in Plant Samples

To determine the P concentration in the collected plant specimens, the samples were first dehydrated to a constant mass in a hot air oven at 60 °C. Subsequent analysis was performed using the vanadomolybdo phosphoric acid colorimetric technique [28,29]. The digestion process was initiated by combining one gram of dried tissue with 10 mL of concentrated HNO_3_ and allowing it to react overnight. This was followed by the addition of 10 mL of a tri-acid mixture (HNO_3_:H_2_SO_4_: HClO_4_, 9:4:1 *v*/*v*) and thorough homogenization. The mixture was then heated on a hot plate, starting at 100 °C for one hour with a subsequent temperature upto 200 °C until the volume was reduced to 2–3 mL and the solution became colorless. After cooling, the digestate was diluted with 10 mL of dilute HCl, filtered through Whatman No. 42 filter paper, and the filtrate was brought to a final volume of 100 mL with distilled water. For color development, a 5 mL aliquot of the digested sample was reacted with 10 mL of vanadomolybdate reagent (Merck, Darmstadt, Germany) for 30 min. The absorbance of the resultant complex was measured at 420 nm using an Analytikjena Specord-200 UV/Vis spectrophotometer (Analytik Jena GmbH+Co. KG, Jena, Germany). Quantification was achieved using a standard curve generated from a known phosphate solution (0.2195 g KH_2_PO_4_ in 500 mL water, acidified with 25 mL of 7N H_2_SO_4_ and diluted to 1 L).

### 2.4. Soil Physico-Chemical, Enzymes, and Microbial Properties

#### Acid (AcP) and Alkaline (AkP) Phosphatase Activity and Soil Microbial Biomass Carbon (MBC)

The phosphatase activities of acid phosphatase (AcP) and alkaline phosphatase (AkP) in soil samples were calculated according to the procedure described by Tabatabai and Bremner [30]. The assay utilized p-nitrophenyl phosphate (pNPP) as the substrate, and results are expressed as micrograms of p-nitrophenol (pNP) generated per gram of soil per hour. For the assay, 1 g of soil was placed into a 50 mL flask, and 0.2 mL of toluene was added, followed by 4 mL of modified universal buffer (MUB) adjusted to pH 6.5 for AcP or pH 11 for AkP, and 1 mL of a 0.05 M pNPP solution. After swirling for one minute, the flasks were incubated at 37 °C for one hour. The reaction was terminated by adding 1 mL of 0.5 M CaCl_2_ and 4 mL of 0.5 M NaOH. The resulting yellow color indicates the p-nitrophenol liberation and was measured spectrophotometrically (Analytikjena Specord-200, Jena, Germany) at a wavelength of 389 nm. A standard curve was prepared with p-nitrophenol standards (0–50 µg mL^−1^) to interpolate the quantity of pNP released in the samples. Microbial biomass carbon (MBC) was assessed via the chloroform fumigation extraction (CFE) technique. The soil moisture content was determined by oven-drying 10 g of moist soil at 105 °C for 24 h. For the CFE procedure, two sets of 3 g soil samples were prepared. One set remained unfumigated, while the other was fumigated with ethanol-free chloroform for 24 h in a vacuum desiccator maintained in darkness. Both fumigated and unfumigated samples were subsequently extracted with 25 mL of 0.5 M K_2_SO_4_. The total organic carbon (TOC) in these extracts was quantified using the dichromate oxidation method [31]. The MBC was calculated by multiplying the difference in extractable organic carbon between the fumigated and unfumigated samples by a factor of 2.64 [32] and is reported as micrograms per gram of dry soil.

### 2.5. Chlorophyll Fluorescence Imaging

Rice plant leaves from the second leaf from the top were collected in trays covered with a moist black cloth. Chlorophyll fluorescence readings were obtained after 30 min of dark incubation for each plant leaf using an imaging fluorometer (Imaging PAM-MAXI-version, Heinz-Walz GmbH, Effeltrich, Germany). This technique, suggested by van Kooten and Snel (1990), was used to measure the minimum fluorescence (F_o_), maximum fluorescence (F_m_), quantum yield of PSII, and F_v/_F_m_, the maximal PSII quantum yield in each leaf sample [33].

### 2.6. Root Scanning

Rice plant roots were collected from each treatment group after 30 d and placed in a Falcon tube containing water. Plastic forceps were used to arrange the scanner roots. This makes it possible to organize the roots in a manner that minimizes their crossing and overlap. The roots were floated in water in acrylic trays on a scanner (Epson Perfection V 700 Photo). Root morphology measurements were performed using Regent Instruments, Inc. Ltd., Quebec, QC, Canada, and Win-RHIZO Programme V. 2009 c 32-bit software was used for analysis and to generate data on the total root length (cm), projected root area (cm^2^), surface root area (cm^2^), root volume (cm^3^), and number of root tips.

### 2.7. Statistical Analysis

All data were analyzed using a factorial analysis of variance (ANOVA) appropriate for a 3 × 2 × 6 CRD factorial design to assess the main effects and interactions among P level, AMF treatment, and rice variety. Treatment means were separated using Duncan’s Multiple Range Test (DMRT) at *p* < 0.05 using the Web-Based Agricultural Statistical Software Package (WASP 2.0) developed by the ICAR-Central Coastal Agricultural Research Institute, Goa (www.ccari.res.in/waspnew.html; https://ccari.res.in/wasp2.0/index.php; https://ccari.res.in/waspnew.html, accessed on 15 May 2022).

### 2.8. P Transporters (OsPT1–OsPT13) Gene Expression

#### 2.8.1. Plant Material

The soil around the roots was removed by washing the roots. Samples of rice plant roots and shoots from each treatment were frozen in liquid nitrogen and stored at −80 °C for further study.

#### 2.8.2. RNA Isolation and cDNA Synthesis

Root samples (100 mg) were placed in a frozen mortar and pestle and ground thoroughly with liquid N_2_ for RNA isolation. Total RNA was extracted from the root samples of each experimental treatment using a QIAwave RNA Mini Kit^®^ RNase-free (Cat. No. 74536, Qiagen, Hilden, Germany), and extracted RNA from all samples of each treatment was purified using the QIAwave RNA Mini Kit^®^ kit (Qiagen) (Cat No. 74904) according to the manufacturer’s instructions and stored at −80 °C for cDNA synthesis [23]. Purity and concentration of RNA samples were measured using the Thermo Scientific NanoDrop™ 1000 Spectrophotometer (Thermo Fisher Scientific, Waltham, MA, USA) and 1.5% agarose gel. RNA samples with different concentrations (ng/µL) and A_260/280_ ratios were used in subsequent experiments (Supplementary Tables S1 and S2). cDNA synthesis for each sample was performed using the qScript cDNA Synthesis Kit (Cat No. 66196094 (Quantabio, MA, USA). DNase I (New England Biolabs, Ipswich, MA, USA) was used to remove DNA contamination from 5 µg of the total RNA. Purified RNA, DNAase, and H_2_O were mixed and kept at 37 °C for 30 min, then 1 µL of 0.5M EDTA was added; placed at 80 °C for 5 min, immediately kept in ice and added 4 µL qScript^TM^ cDNA buffer solution was added, and PCR was run at 25 °C for 10 min, 42 °C for 1 h, and 80 °C for 5 min. After PCR, all samples were stored under cooling conditions. First-strand cDNA synthesis was performed using 2 µg of DNase I-treated RNA. The cDNA solution was diluted with nuclease-free water, and aliquots were stored at −20 °C until use in RT-qPCR [34]. All experiments were performed in strict accordance with the manufacturer’s instructions.

#### 2.8.3. Primers of P Transporter Genes

All gene-specific primer sequences of P transporters (*OsPT1*–*OsPT13*) used for RT–qPCR are listed in Supplementary Table S3 [23]. Primers were synthesized by AgriGenome Pvt. Ltd. (Hyderabad, India) and Integrated DNA Technologies (IDT), Gurgaon, India.

#### 2.8.4. P Transporters (*OsPT1*–*OsPT13*) Gene Expression by RT–qPCR

Real-time quantitative PCR (qPCR) was performed using TB Green Premix Ex Taq II (Tli RNase H Plus), TAKARA BIO Inc., Shiga, Japan (Cat No. RR820A) on BIO-RAD Real-Time PCR, Bio-Rad Laboratories, Inc., Hercules, CA, USA [35,36] was run according to the manufacturer’s instructions. Amplifications were carried out in 10 μL reaction solutions (Premixed TB green: 5 µL, forward primer: 0.5 µL, reverse primer: 0.5 µL, cDNA: 1 µL, dH_2_O: 3 µL) and RT-PCR program is 95 °C for 30 s; 95 °C for 15 s; 60 °C for 10 s; 72 °C for 30 s; 40 cycles and melting curve analysis: 95 °C for 5 s; 65 °C for 5 s; 95 °C for 5 s; for quantification of cycle (Cq). The rice 18S RNA gene (forward primer: 5′—CTA CGT CCC TGC CCTT TGTAC—3′, reverse primer: 5′—ACA CTT CACC GGAC CAT TCAA—3′) was used to normalize gene expression [37], and the results were compared to the P susceptible variety IR 36, each of having biological and technical replicates. The specificity of the reaction was analyzed using a melting curve analysis. The relative transcript level of the mRNA was determined by ∆∆^CT^ values in comparison with IR36, keeping the expression value of the gene in IR36 at one. For analysis at different time points, the cycle threshold (Ct) value for the control samples was taken as one, and the fold change at other time points was calculated using a similar method [23,38].

## 3. Results and Discussion

### 3.1. Effect of AMF Colonization and Sporulation in Different Rice Varieties Under Different P Conditions

AMF increase the accessibility of soil nutrients and phosphate through extra-radical mycelium formation, which operates as a functional activity in plants [39]. Proving the proposed accessibility of P through mycorrhiza, Chareesri et al. [40] reported that AMF inoculum *viz*. *F. mosseae*, *R. irregularis C. claroideum*, *G. microaggregatum*, and *F. geosporus* (10 AMF spores per 100 g soil) application in rice enhanced the colonization of AMF by 16.00 ± 3.30% as compared to uninoculated control (7.0 ± 1.60%). Similarly, Sahoo et al. [39] observed that the AMF intervention increased AMF colonization and sporulation by 14–56% and 32–69%, respectively, compared to uninoculated control after 60 days of rice planting. Maiti et al. ([14] documented that the potential of AMF to increase upland rice yield by increasing AMF activity and facilitating plant P acquisition. The combination of maize and horse gram crops in a rotational pattern, inoculated with AM fungi, increased AMF colonization by 22.7 to 42.7% under upland conditions. Vallino et al. [12] reported that 13 rice varieties colonized by AMF with a range of 4.0–28% under aerobic conditions. The above findings clearly indicated that mycorrhizal root colonization is important for plant nutrient uptake and that colonization can be improved by external application of AMF, but it may vary from variety to variety. In the present study, AMF colonization was 15.0–24.0% lower in high-P soils compared to low-P soils. Among the varieties, CR Dhan 204, 205, and 207 had significantly higher AMF colonization (50.19–63.63%) and sporulation (24.29–30.28 spores 50 g soil^−1^) in low and medium soil P as compared to other varieties (Table 1; Supplementary Figure S4.1–S4.3). This finding indicates that colonization and sporulation of AMF vary from variety to variety, irrespective of Soil P; however, the increasing Soil P gradually decreases colonization and sporulation regardless of the rice variety.

### 3.2. Influence of AMF Inoculation on P Uptake in Different Rice Varieties Under Low, Medium, and High Soil P Conditions

**Figure 1:**
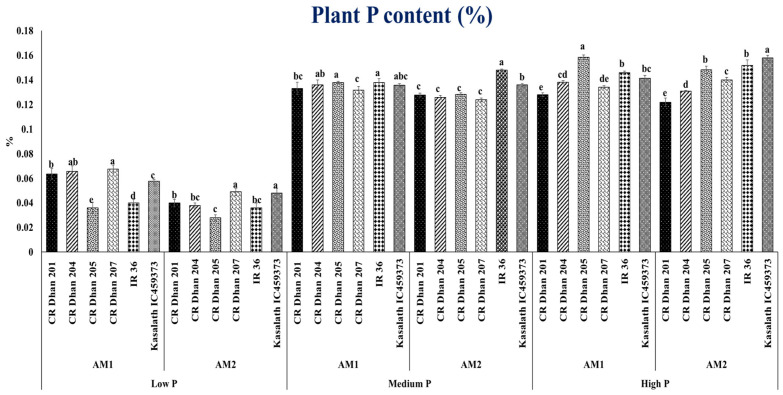
Effect of AMF inoculation on P uptake in different rice varieties. (AM1: with AM fungi; AM2: without AM fungi; lowercase letters represent significant variations among the data at *p* < 0.05).

Phosphorus is an essential macronutrient required by plants for healthy growth and development; however, fixation restricts the accessibility. P content would be around 0.05 and 0.5% of the dry weight of the entire plant [41]. Although the amount of P in soils is 2000 times greater than that in plants, the majority of P is inaccessible to plants because of fixation by Mg, Al, Fe, Ca, etc. Thus, P deficiency affects plants in most agricultural soils [42]. In rice, plant biomass and grain yield have been severely affected by declining P availability [43]. To overcome problems and difficulties of P management, AMF are one of the key components of the soil microbiome that promote the P translocation in the rice plant [8,9,10,11,44,45]. The effect of AMF in upland rice indicated that increasing AMF activity increases the plant P uptake and AM fungal colonization (*Glomus* and *Acaulospora*) by +11.2 to +23.7% and grain yield by +25.7 to +34.3% with the maize-horse-gram rotation [14]. In our present study, there was no significant difference in P content between AMF-treated and untreated CR Dhan 207 and Kasalath IC459373 varieties under medium soil P conditions (Figure 1), but the CR Dhan 207 variety demonstrated 50.22% higher P uptake than CR Dhan 205 (without AMF inoculation) under low-P soil (Figure 1). In all the rice varieties treated with AMF had significantly higher P content than the uninoculated control under low-P soil (Figure 1); however, CR Dhan 207, CR Dhan 204, and CR Dhan 201 showed significantly higher uptake of P than CR Dhan 205. The beneficial effects from AMF varies one variety to another.

### 3.3. AMF Inoculation on Biomass Production in Different Rice Varieties Under Low, Medium, and High Soil P Conditions

**Figure 2:**
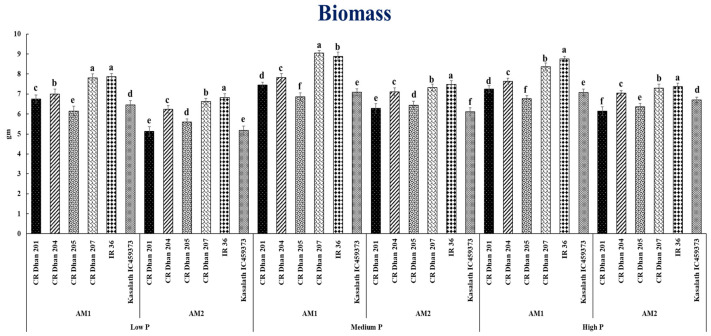
Effect of AMF inoculation on biomass production in different rice varieties. (AM1: with AM fungi; AM2: without AM fungi; lowercase letters represent significant variations among the data at *p* < 0.05).

Numerous studies have shown that AMF inoculation and interactions in the soil are the major responses that maintain the highest plant growth and increase their biomass under various stress conditions [46,47,48,49,50]. Njaramanana et al. [51] conducted an experiment to understand the influence of (*R. irregularis*) on four promoted upland rice varieties without P fertilizer supply in Madagascar and observed enhanced rice growth, P uptake, grain yield (28.00%), grain N (30.00%), and grain P (39.00%) compared to the uninoculated control. They also reported that AMF seed coating inoculation at the field scale greatly enhanced upland rice plant performance in a low soil P environment, even in the absence of P fertilization. In the present study, all AMF-treated aerobic rice varieties showed significantly higher plant biomass under different soil P conditions (Figure 2). In low-P soil, there was a significant percentage of enhancement of plant biomass compared to the uninoculated control. However, plant growth was also enhanced in medium and high soil, which might be due to some other beneficial bacteria associated with AMF spores, as well as to the higher dosage of phosphorus fertilizer application. Many studies have reported that mycorrhizal spore-associated bacteria play an important role in promoting plant growth in crops.

### 3.4. Effect of AMF Inoculation on Acid and Alkaline Phosphatase Activity in Different Rice Varieties Under Low, Medium, and High Available Soil P Conditions

**Figure 3:**
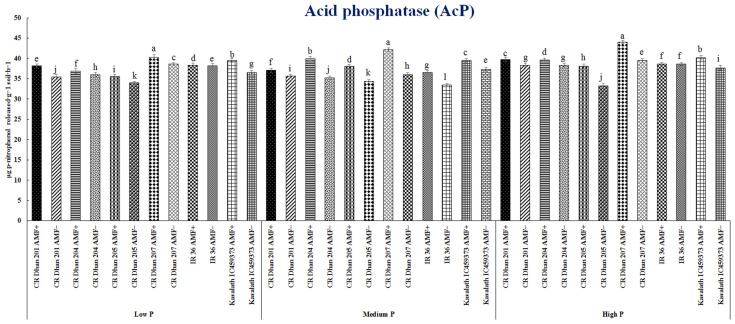
Effect of AMF inoculation on AcP activity in different rice varieties. (AMF+: with AM fungi; AMF−: without AM fungi; lowercase letters represent significant variations among the data at *p* < 0.05).

**Figure 4:**
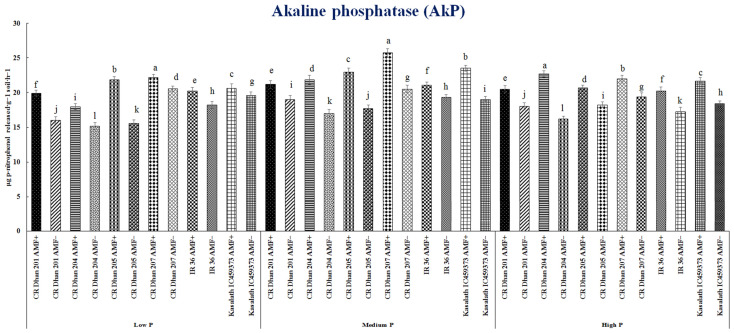
Effect of AMF inoculation on AkP activity in different rice varieties. (AMF+: with AM fungi; AMF−: without AM fungi; lowercase letters represent significant variations among the data at *p* < 0.05).

Acid phosphatase (AcP) and alkaline phosphatase (AKP) are crucial for P metabolism and nutrient uptake in plants [52]. These enzymes are responsible for the release of phosphate (P*i*) from various organic P molecules, thereby allowing plants to absorb and use Pi [53,54]. In rice, several factors, such as genotype, developmental stage, and environmental conditions, influence AcP activity [33]. In low-P soils, some plants use organic phosphate to secrete AcP from their roots [55], and different rice varieties may have varying levels of AcP and AkP activity based on their genetic makeup and adaptation to different environments. The present study demonstrated that different rice varieties, with and without AMF inoculation, can affect P uptake by enhancing and releasing phosphatase enzymes under various P conditions. All rice varieties treated with AMF had significantly higher AcP in low-P soil than the uninoculated control. However, AMF-treated CR Dhan 207 and CR Dhan 204 showed higher AcP at all three levels of soil P (Figure 3). In low and medium soil P, CR Dhan 207 and CR Dhan 204 with AMF increased AkP activity by 28.7–34.03% compared to other varieties (Figure 4).

### 3.5. Effectiveness of AMF Inoculation on Soil Microbial Biomass Carbon (MBC) in Different Rice Varieties Under Low, Medium, and High Soil P

**Figure 5:**
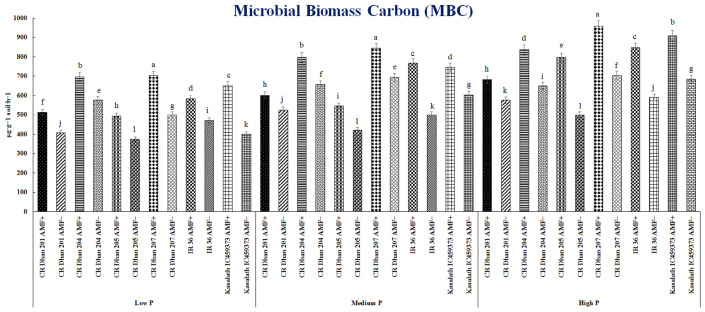
Effect of AMF inoculation on MBC in different rice varieties. (AMF+: with AM fungi; AMF−: without AM fungi; lowercase letters represent significant variations among the data at *p* < 0.05).

Soil microbial biomass carbon (MBC), a microbiological component of soil organic matter, is considered an indicator of microbial activity because of its rapid response to conditions that may affect soil organic matter [56]. Jabborova et al. [57] reported that AMF inoculated with biochar could improve soil attributes, enhance microbial activity, and promote plant growth. Similarly, the application of biochar along with AMF (*R. irregularis*) in rice (*O. sativa*) significantly enhanced soil properties and plant growth [58]. The root mycorrhizal colonization rate was substantially linked to available K, available P, total N, MBC, and microbial biomass nitrogen (MBN). The results indicate that soil MBC and MBN were enhanced by interactions between biochar and AMF, on nutrient availability. This study aimed to comprehend the MBC activity and effects of AMF inoculation, with and without different aerobic rice and check varieties, at various P levels. The results showed that the AMF-inoculated CR Dhan 207 variety resulted in 46.74%, 49.99%, and 47.79% higher MBC than the other rice varieties in low-, medium-, and high-P soils, respectively. Compared to un-inoculated varieties, all rice varieties grown in high-P soil, particularly CR Dhan 207, CR Dhan 204, and Kasalath IC459373, had considerably higher MBC (Figure 5).

### 3.6. Influence of AMF Inoculation on Chlorophyll Fluorescence in Different Rice Varieties Under Low, Medium, and High Available Soil P Conditions

**Figure 6:**
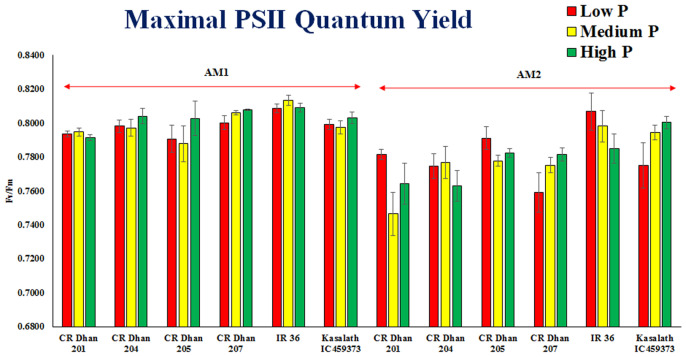
Chlorophyll fluorescence results in different rice varieties under low, medium, and high available soil P conditions. [AM1: with AM fungi; AM2: without AM fungi; F_o_—minimum fluorescence when all the reaction centers are open; F_m_—maximum fluorescence when all the reaction centers are closed; F_v_—variable fluorescence (F_m_ − F_o_); F_v_/F_m_ = (1 − F_o_/F_m_)].

Plant tolerance to stress may be significantly influenced by the effectiveness of the photosynthetic processes. When growing conditions are unfavorable, plants can sense them and initiate an internal response before external symptoms appear [59]. Chlorophyll fluorescence analysis is a reliable method for determining the maximum efficiency of photosystem II (PSII) (F_v_/F_m_) photochemical functions in rice plants under stress [60]. According to Chareesri et al. [40], under drought conditions, plants with higher AMF colonization show higher stomatal conductance and chlorophyll fluorescence than plants with lower colonization. It is possible that AMF symbiosis stimulated photosynthesis at the sink in plants with higher AMF colonization, as evidenced by the plants’ slightly but considerably higher chlorophyll fluorescence [61]. The beneficial effects of AMF on chlorophyll fluorescence were found that mycorrhizal rice plants showed higher chlorophyll fluorescence than non-mycorrhizal plants under stress. The study was conducted to determine PSII quantum yield efficacy under different P levels, with and without AMF treatment in different rice varieties, *viz*. CR Dhan 201, 204, 205, 207, Kasalath IC459373, and IR36 (Supplementary Figure S5). Figure 6 represents chlorophyll fluorescence scanning imaging in the PAM–MAXI version, Heinz Walz GmbH, and records F_o_, F_m_, and F_v_/F_m_. Overall, the results showed that PSII quantum yield was higher in the AMF plants than in the uninoculated control (Figure 6).

### 3.7. Impact of AMF Inoculation on Improvement of Root Growth and Architecture Parameters in Different Rice Varieties Under Different Soil Available P Conditions

**Figure 7:**
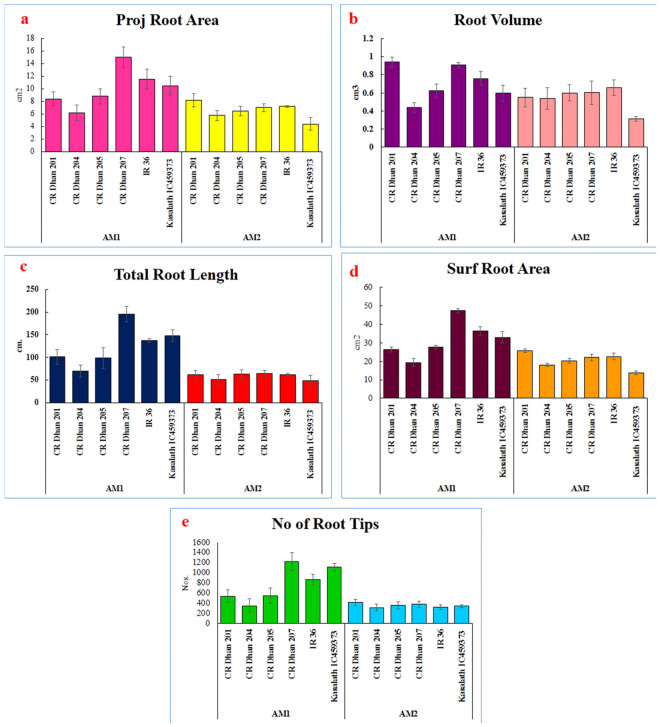
Root architecture of different rice varieties under low soil available P conditions with and without AMF treatment. (**a**) projected root area, (**b**) root volume, (**c**) total root length, (**d**) surface root area, and (**e**) number of root tips (AM1: with AM fungi; AM2: without AM fungi).

Arbuscular mycorrhizal symbiosis has been found to significantly improve root architecture in rice plants [62,63]. AMF enhances the growth of fine roots and lateral root branching [64]. Branching expands the root surface area, thereby enhancing water and nutrient absorption. Root length and density often increase after AMF inoculation [65]. This study aimed to understand changes in root architecture in various rice varieties at different P levels, both with and without AMF treatment, after 20 d (Supplementary Figure S6.1). Under low-P conditions, CR Dhan 207 showed 64.09, 59.13, 59.13, 51.43, and 71.22% higher total root length (cm), projected root area (cm^2^), surface root area (cm^2^), root volume (cm^3^), and number of root tips, respectively, compared to uninoculated plants (Figure 7; Supplementary Figure S6.2).

**Figure 8:**
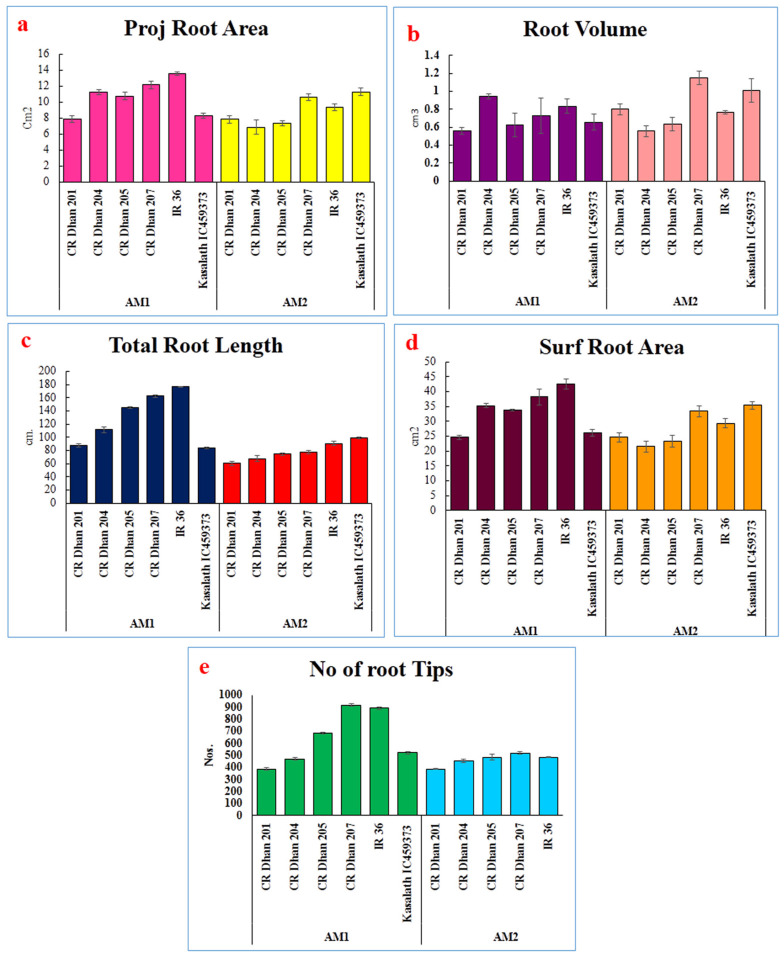
Root architecture of different rice varieties under medium soil available P condition with and without AMF treatment. (**a**) Projected root area, (**b**) root volume, (**c**) total root length, (**d**) surface root area, and (**e**) number of root tips (AM1: with AM fungi; AM2: without AM fungi).

In medium-P soil with AMF, IR 36 showed 52.78, 42.01, and 42.01% more total root length (cm), projected root area (cm^2^), and surf root area (cm^2^), respectively; the CR Dhan 204 variety showed 34.00 and 33.47% more average root diameter and root volume, respectively; and CR Dhan 207 showed 57.75% more root tips (Figure 8; Supplementary Figure S6.3).

**Figure 9:**
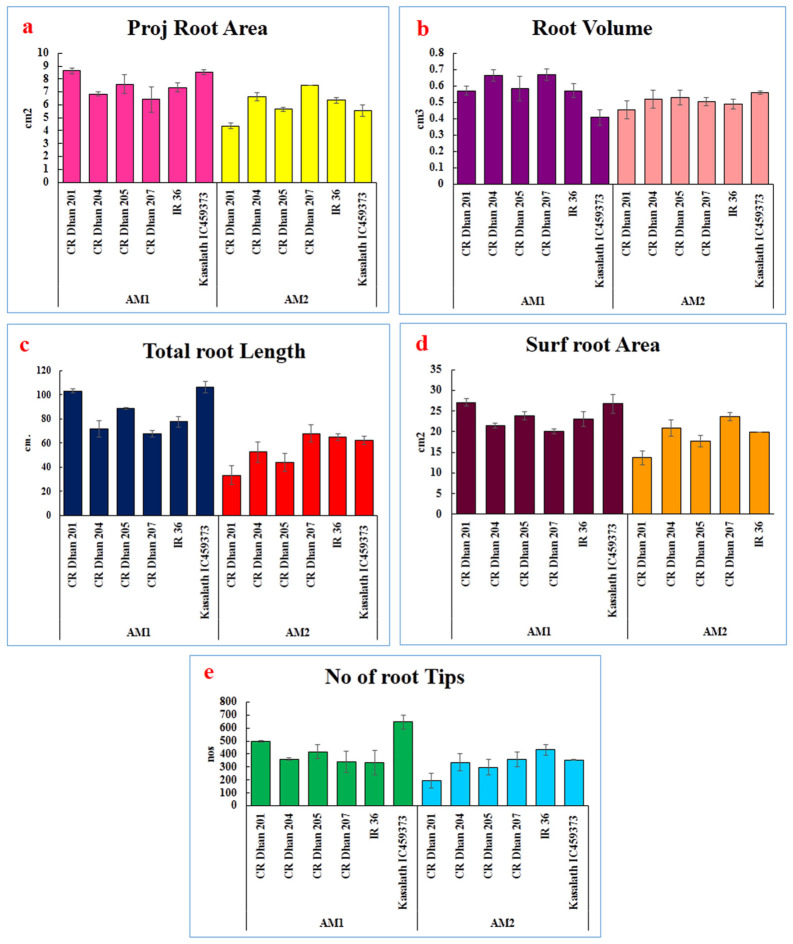
Root architecture of different rice varieties under high soil available P conditions with and without AMF treatment. (**a**) projected root area, (**b**) root volume, (**c**) total root length, (**d**) surface root area, (**e**) number of root tips (AM1: with AM fungi; AM2: without AM fungi).

However, with AMF inoculation in high-P soil, Kasalath showed 47.98 and 36.65% more root tips and total root length, respectively; CR Dhan 201 showed 34.66, 25.74, and 11.85% more projected root area, surface root area, and root volume, respectively (Figure 9; Supplementary Figure S6.4).

AMF inoculation in different rice varieties significantly increased the total root length (cm), surface root area (cm^2^), projected root area (cm^2^), root volume (cm^3^), and number of root tips under low soil P compared to the uninoculated control. In general, there was approximately a 33.0–55.0% enhancement in root architecture with AMF inoculation in all the varieties at low and medium available soil P levels (Figure 7, Figure 8 and Figure 9). The significant correlation coefficient observed between enhanced root architecture traits and the differential expression of *OsPT* genes highlights a complex, bidirectional regulatory feedback loop within the rhizosphere. It is critical to differentiate between primary mycorrhizal-induced signaling and secondary physiological homeostatic adjustments. Among the profiled transporters, *OsPT11* represents a classically documented, symbiosis-specific marker directly driven by conserved AMF signaling cascades upon functional interface establishment [15,20]. Conversely, the transcriptomic modulations observed across the majority of the non-symbiotic, direct-pathway *OsPT* transporters (such as *OsPT1*, *OsPT2*, and *OsPT6*) are highly likely secondary feedback responses rather than directly driven by the fungus.

Mycorrhizal colonization radically alters the plant’s physiological landscape by significantly improving internal orthophosphate (Pi) allocation and modifying root endogenous hormonal profiling (such as auxin and strigolactone balance), which fundamentally reshapes overall root architecture and plant fitness [66]. As internal cellular Pi pools replenish via the highly efficient mycorrhizal uptake pathway, the plant dynamically downregulates or tunes its high-affinity direct root epidermal transporters via systemic P-sensing networks to avoid cellular toxicity and balance energy expenditure. Therefore, the observed transcriptional shifts in non-symbiotic *OsPT* genes function as a fine-tuned secondary feedback mechanism, coordinated by the host plant, to match its improved internal phosphorus status and enhanced root absorptive surface area.

### 3.8. Relative Expression of P Transporters (OsPT1–OsPT13)

Rice plant roots absorb P from the soil as inorganic phosphate (Pi) [67]. Most pi in native soils can be found as metal ion salts or pi-esters, which are not easily utilizable by plants [68]. Pi concentrations in soil solutions are typically low (~1–10 µM) due to fixation and low solubility, while cytoplasmic Pi concentrations in plant cells generally range from 1 to 5 mM [69]. In aerobic rice cultivation, P fixation and its availability to plants are major challenges, which limit the rice production. To overcome this high concentration difference and enhance the availability of soil P, plants have established a variety of high/low-affinity Pi-transporter (PT) transporter-based processes with AMF intervention [20,23,70,71,72]. Rice plant growth and development require the coordinated activation of numerous distinct Pi transport mechanisms, based on increasing physiological and cellular evidence. It is evident that tissues other than roots and leaves also use transporters for Pi transport.

Phosphate Transporters (PTs) have been identified in several plant species and belong to two major gene families, *Pht1* and *Pht2*. Multiple PTs belong to the *Pht1* family in many plant species. Various physiological and biochemical processes involve the phosphate transporter genes *OsPT1*–*OsPT13*. *OsPT1* is constitutively expressed in roots and is involved in phosphate uptake from the soil into the root epidermis and hairs under phosphate-deficient conditions. It is clear that this gene is crucial for Pi acquisition under these conditions [17,18,73]. *OsPT2* plays crucial roles in Pi root-to-shoot translocation and Pi homeostasis in the shoots [74,75]. It is extensively expressed in both the stele cells of rice roots and shoots of rice. In addition, it regulates phosphate homeostasis and sensing. Pi accumulation and transfer in rice are facilitated by the low-affinity phosphate transporter gene *OsPT2* [76,77]. *OsPT3*, which aids in phosphate absorption from the soil, is expressed in the root epidermis and root hair. It is crucial in low-phosphate environments and helps rice grow [17,18]. Similarly, *OsPT4* participates in phosphate uptake and is expressed in the cortex and root epidermis. It participates in phosphate homeostasis and is activated during phosphate deficiency. Rice functions as a Pi influx transporter that is involved in Pi absorption, seed germination, and Pi translocation [17]. *OsPT5*, which is involved in phosphate uptake. It contributes to phosphate homeostasis and is affected by phosphate deficits. *OsPT6* plays a role in phosphate uptake and is expressed in the cortex and root epidermis of plants. It is transported by phosphate deficiency and enhances phosphate uptake and accumulation in transgenic rice plants [75]. In transgenic rice plants, *OsPT6* increased phosphate uptake and accumulation [17,18]. *OsPT7* participates in phosphate uptake and is expressed in the cortex and root epidermis of rice plants. It participates in phosphate homeostasis and is activated in phosphate-poor environments [17,78]. *OsPT8* is abundantly expressed in the rice root stele and shoot leaves, where it is crucial for Pi root-to-shoot translocation and shoot Pi homeostasis [79]. The root epidermis and cortex contain *OsPT8*, which plays an important role in phosphate uptake. In rice, phosphate homeostasis is regulated by the phosphate transporter gene *OsPht8* [17,18,80,81]. It contributes to phosphate homeostasis and is induced by phosphate deficiency. Plant growth and disease resistance are regulated by *OsPT8* [82,83]. The root epidermis and cortex express *OsPT9*, which is involved in phosphate uptake [15]. It plays a role in phosphate homeostasis and is upregulated under phosphate-deficient conditions [84]. Low Pi status induces *OsPT9* gene expression, its localization in several plant tissues, and enhancement of Pi transport characteristics [82]. The root epidermis and cortex express *OsPT10*, facilitating phosphate uptake. It contributes to phosphate homeostasis and is induced by phosphate deficiency in plants. A low Pi status induces *OsPT10* gene expression and localization in several plant tissues, and enhances Pi transport [82,85]. The root epidermis and cortex express *OsPT11*, which is involved in phosphate uptake. It is caused by phosphate shortage and helps maintain phosphate homeostasis. *OsPT11* participates in fungal symbiosis, known as arbuscular mycorrhiza [86]. *OsPT12* is expressed in the cortex and root epidermis and aids in phosphate uptake. It is upregulated under phosphate-deficient conditions and contributes to the maintenance of phosphate homeostasis [87]. The root epidermis and cortex express *OsPT13*, which is involved in phosphate uptake [84]. It contributes to phosphate homeostasis and is induced by phosphate deficiency [71]. *OsPT13* is essential for AM symbiosis and phosphate absorption [19]. *OsPT13* may have an insignificant effect on symbiotic Pi absorption. For effective phosphate acquisition and utilization, particularly in phosphate-limited situations, rice requires these phosphate transporter genes [88]. According to Yang et al. [20], *OsPT11* may be responsible for both AM development and symbiotic phosphate uptake, whereas *OsPT13* may serve as a sensor for determining the proper phosphate level for arbuscule development [89].

**Figure 10:**
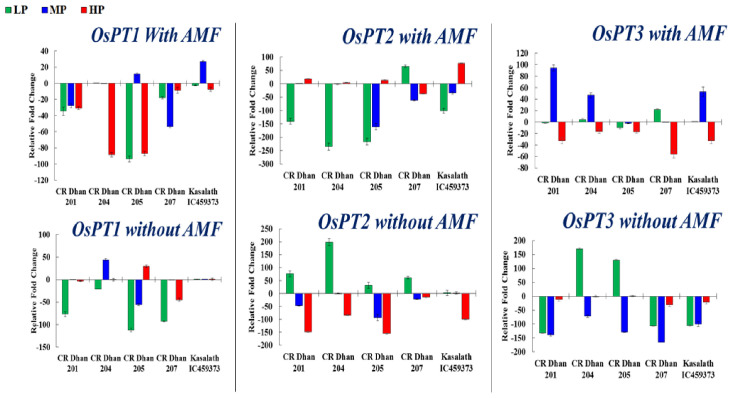
Relative expression of P transporters (*OsPT1–OsPT3*) in different rice varieties under low, medium, and high soil available P conditions with and without AMF application.

**Figure 11:**
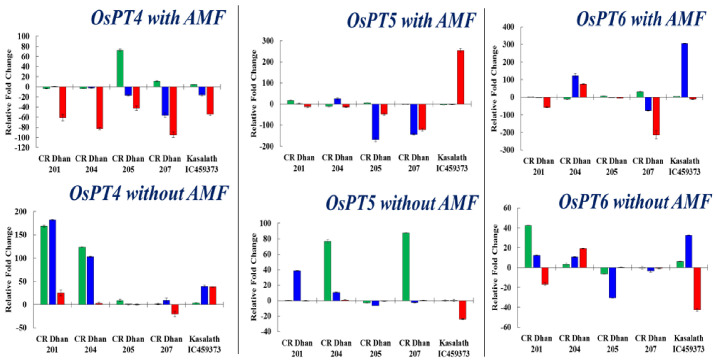
Relative expression of P transporters (*OsPT4–OsPT6*) in different rice varieties under low, medium, and high soil available P conditions with and without AMF application. Green: low-P; Blue: medium-P; Red: high-P.

**Figure 12:**
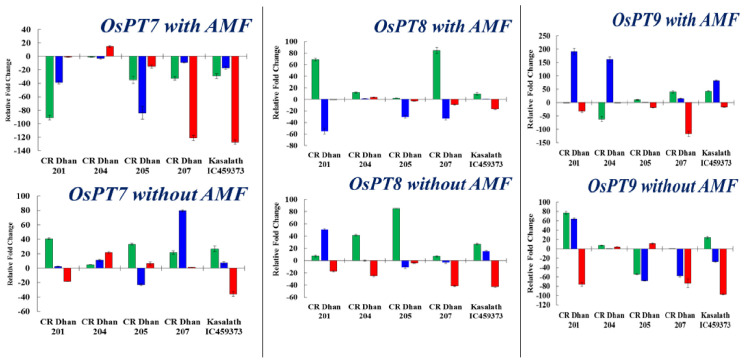
Relative expression of P transporters (*OsPT7–OsPT9*) in different rice varieties under low, medium, and high soil available P conditions with and without AMF application.

**Figure 13:**
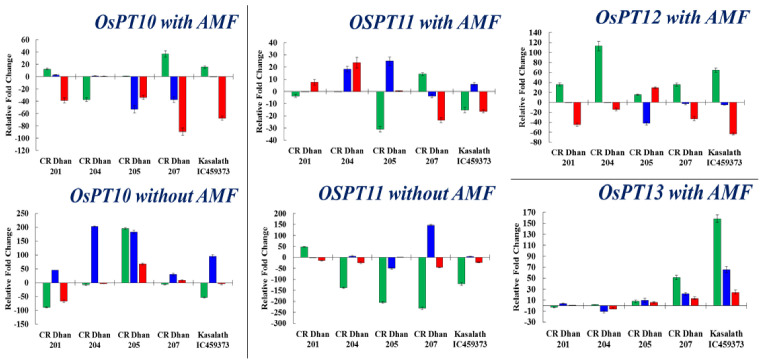
Relative expression of P transporters (*OsPT10–OsPT13*) in different rice varieties under low, medium, and high soil available P conditions with and without AMF application.

Figure 10, Figure 11, Figure 12 and Figure 13 indicate the relative fold changes in the expression of *OsPT* genes (*OsPT1* to *OsPT13*) in the varieties *viz*. CR Dhan 201, CR Dhan 204, CR Dhan 205, CR Dhan 207, IR 36 (P susceptible), and Kasalath IC459373 (P tolerant), which were subjected to treatments with and without application of AMF at low, medium, and high-P levels. *OsPT1* expression was 27.14- and 11.35-fold higher in Kasalath IC459373 and CR Dhan 205, respectively, under medium-P conditions with AMF inoculation (Figure 10). According to previous reports, *OsPHR2*-mediated accumulation of Pi in rice shoots is caused by *OsPT2*, a low-affinity Pi transporter in rice [90]. According to Li et al. [76,77], the constitutive overexpression of *OsPT2* may negatively affect rice seedling growth under high Pi conditions. In this study, *OsPT2* was found to be downregulated by AMF intervention in CR Dhan 201, CR Dhan 204, and CR Dhan 205, whereas CR Dhan 207 showed higher gene expression with a 4.72- fold increase in low-P soil. *OsPT3* expression under low-P conditions increased 21.90- fold in CR Dhan 207 after AMF application. All varieties *OsPT3* were found to be downregulated without the application of AMF, but AMF treatment had an influence on *OsPT3* upregulation under medium-P conditions in CR Dhan 201 (94.15- fold), CR Dhan 204 (46.71- fold), and Kasalath IC459373 (52.65- fold) (Figure 10).

AMF treatment had a positive impact on the expression of *OsPT4* in CR Dhan 205, CR Dhan 207, and Kasalath IC459373, with an increase of up to 63.46-, 10.12-, and 1.45-fold, respectively, but it was downregulated in CR Dhan 201 and CR Dhan 204 (Figure 11). The expression of *OsPT5* was observed to increase by 254.24 and 16.86-fold in Kasalath IC459373 and CR Dhan 201, respectively, following AMF inoculation under high and low-P conditions, as determined by relative fold-change calculations (Figure 11). Under low-P soil conditions, the findings observed that the CR Dhan 207 gene was upregulated by a relative fold change of 30.08 in the expression of the *OsPT6* gene with the application of AMF (Figure 11).

Under high-P soil conditions, the CR Dhan 204 gene was upregulated by a relative fold change of 14.89 in the expression of the *OsPT7* gene with AMF application (Figure 12). Interestingly, it was observed that in low-P soil, the rice varieties CR Dhan 201 and CR Dhan 207 had their expression of *OsPT8* increased by more than 61.39- and 77.50-fold, respectively, with AMF inoculation. However, with AMF inoculation, the expression of *OsPT8* decreased in CR Dhan 204, CR Dhan 205, and Kasalath IC459373 by 29.42-, 82.87-, and 17.07-fold, respectively, in low-P soil (Figure 12). The rice varieties *viz*. CR Dhan 205, CR Dhan 207, and Kasalath IC459373 were affected by *OsPT9* gene expression in low-P soil with AMF by 10.85-, 40.19-, and 19.25-fold, respectively. Under medium-P soil with AMF, *OsPT9* gene expression increased by 127.61-, 160.99-, 14.86-, and 82.17-fold in rice varieties *viz*. CR Dhan 201, CR Dhan 204, CR Dhan 207, and Kasalath IC459373, respectively (Figure 12).

AMF treatment had a positive influence on the expression of the *OsPT10* gene in CR Dhan 201, CR Dhan 207, and Kasalath IC459373, with an increase of up to 12.42-, 36.90-, and 15.48-fold under low-P soil (Figure 13). In the case of the arbuscular mycorrhizal symbiotic relationship, the *O. sativa* phosphate transporter (*OsPT*) gene *OsPT11* is particularly promoted when *G. intraradices* colonizes the roots—*OsPT11* activation occurs independently of the plant’s nutritional state and the amount of phosphate available in the rhizosphere. The research showed that CR Dhan 207 showed significant *OsPT11* gene upregulation, with an increase of 14.17-fold in low-P soil. Similarly, in medium-P soil, the *OsPT11* gene was upregulated 18.27-, 24.80, and 5.85-fold, respectively, in CR Dhan 204, CR Dhan 205, and Kasalath IC459373. *OsPT11* gene expression was increased 23.52- fold by CR Dhan 204 in the high-P soil (Figure 13). No gene expression was observed in *OsPT12* or *OsPT13* without AMF inoculation (Figure 13). The results showed that all rice varieties treated with AMF, including CR Dhan 201, CR Dhan 204, CR Dhan 205, CR Dhan 207, and Kasalath IC459373, had *OsPT12* gene regulation of 35.70, 113.01, 15.32, 35.58, and 64.72- fold, respectively, in the low-P soil (Figure 13). Similar results were obtained with CR Dhan 2014, CR Dhan 205, CR Dhan 207, and Kasalath IC459373 in low-P soils, where *OsPT13* gene expression was significantly upregulated by 1.51-, 7.86-, 51.19-, and 158.50- fold, respectively (Figure 13).

Numerous studies have reported that plant species have PTs from two major gene families (*Pht1* and *Pht2*) [64,78,91,92,93]. *OsPht1*;2 and *OsPht1*;6 have different functions and kinetic properties in rice P uptake and translocation of Pi, according to research by Ai et al. [17,18] on the expression patterns of *Pht1* genes in the model cultivar of *Oryza sativa* ssp. Japonica *cv.* Nipponbare. *Pht1* promoter-GUS and RT-PCR results showed that at least eight of the 13 *Pht1* genes (*OsPT1*, *OsPT2*, *OsPT3*, *OsPT4*, *OsPT5*, *OsPT6*, *OsPT7*, and *OsPT8*) were identified in rice roots, and that the majority of these genes were induced by Pi deprivation. Similar findings under Pi starvation: High levels of *OsPT1*, *OsPT2*, *OsPT6*, *OsPT7*, and *OsPT8* expression have been identified in root-shoot junctions and leaves, suggesting that they may be involved in Pi translocation from roots to shoots in rice. They are also likely to play a role in Pi redistribution to young organs during leaf senescence [94].

In this study, the expression of phosphate transporter genes (*OsPT1* to *OsPT13*) was kept constant (unit value) in variety IR36, and the relative fold-change in expression was examined in other varieties. CR Dhan 201, CR Dhan 204, CR Dhan 205, CR Dhan 207, IR 36 (P-susceptible), and Kasalath IC459373 (P-tolerant) treated with and without AMF under three levels of P condition *viz*., low-, medium-, and high-P (Figure 10, Figure 11, Figure 12 and Figure 13). Among all the varieties, out of the 13 *OsPT* genes, 10 *OsPT* genes (*OsPT2*, *OsPT3*, *OsPT4*, *OsPT6*, *OsPT8*, *OsPT9*, *OsPT10*, *OsPT11*, *OsPT12,* and *OsPT13*) were expressed in CR Dhan 207 in conditions of low soil P availability with the inoculation of AMF. P absorption and Pi translocation under low-P conditions were observed for arbuscular mycorrhizal symbiosis in CR Dhan 207, followed by CR Dhan 205, Kasalath IC459373, CR Dhan 201, and CR Dhan 204. The present findings clearly indicate that the involvement of arbuscular mycorrhizal fungi (AMF) in aerobic rice is crucial for P uptake under low and medium-P conditions. AMF also play a key role in enhancing the expression of various phosphate (Pi) transporters in response to different levels of available soil P.

## 4. Conclusions

In summary, our comparative evaluation identifies CR Dhan 207 as a highly responsive priority genotype for aerobic rice production under phosphorus-limited soils. We propose a brief mechanistic hypothesis that the superior performance of CR Dhan 207 is fundamentally driven by enhanced symbiotic signaling efficiency paired with accelerated early-stage arbuscular colonization. This variety is likely characterized by an optimized root exudation profile—specifically involving structural signals like strigolactones—that rapidly recruits and establishes functional interfaces with the inoculated AMF consortium (consisting of *Funneliformis*, *Rhizophagus*, *Glomus*, *Acaulospora*, and *Claroideoglomus* species). This accelerated synchronization effectively orchestrates a dual-acquisition network, combining the mycorrhizal-mediated uptake pathway via *OsPT11* with the fine-tuned modulation of direct epidermal OsPT transporters to optimize physiological phosphorus assimilation. The present study indicates that among the different aerobic varieties, CR Dhan 204, CR Dhan 205, and CR Dhan 207 had significantly higher AMF colonization (50.19–63.63%) and sporulation (24.29–30.28 spores 50 g soil^−1^) under low and medium levels of soil P than other varieties. In all the rice varieties, there was a 33–55% enhancement of root growth parameters in the AMF treatment at low and medium levels of soil P. Under P-deficient soil, all rice varieties treated with AMF recorded significantly higher P uptake (14.87–50.22%) compared to the uninoculated control. A similar trend was observed in plant biomass (CR Dhan 207 and IR36) and soil microbial properties such as AcP, AkP, MBC, and DHA (CR Dhan 207 and CR Dhan 204) compared to the uninoculated control. Among all varieties, most of the phosphate transporter genes (10 genes viz. *OsPT2*, *OsPT3*, *OsPT4*, *OsPT6*, *OsPT8*, *OsPT9*, *OsPT10*, *OsPT11*, *OsPT12*, and *OsPT13*) were expressed in CR Dhan 207 under low available soil P conditions. However, it is essential to contextualize these variety-specific outcomes within the structural boundaries of a controlled pot experiment. Artificial container constraints introduce unique ecological conditions, such as physical root confinement and a simplified microbial matrix, that differ significantly from open agro-ecosystems. Physical root restriction in pots often artificializes localized nutrient-depletion zones, potentially over-activating high-affinity direct transport systems. In dynamic field environments, complex multi-trophic interactions, fluctuating soil moisture gradients, and indigenous microbial competition may alter or buffer the expression patterns of these genes. Therefore, while our pot trial provides a critical mechanistic blueprint of host-symbiont genetic capacity, broad-scale field validation remains an essential future step to confirm the true real-world stability of these variety-specific responses. So, the present study concluded that AMF fungal intervention in aerobic rice can enhance the regulation of P transporter genes and improve P uptake under low to medium-P soil conditions. However, the response varies among rice varieties, indicating the need for further field validation. These findings also emphasize the importance of identifying mycorrhizal-responsive aerobic rice varieties to address P management challenges in P-deficient soils.

## Figures and Tables

**Table 1:** AMF colonization and sporulation in different rice varieties at different soil P.

	Varieties	% of AMF Colonization	AMF Sporulation (Spores 50 g Soil^−1^)
Low P	CR Dhan 201	58.749 ^d^	26.076 ^d^
	CR Dhan 204	62.188 ^ab^	23.664 ^fg^
	CR Dhan 205	63.631 ^a^	30.825 ^a^
	CR Dhan 207	53.197 ^fg^	29.496 ^ab^
	IR 36	56.905 ^e^	30.822 ^a^
	Kasalath IC459373	53.756 ^f^	26.076 ^d^
Medium P	CR Dhan 201	56.169 ^e^	25.573 ^de^
	CR Dhan 204	60.037 ^cd^	24.290 ^ef^
	CR Dhan 205	61.491 ^bc^	27.749 ^c^
	CR Dhan 207	50.197 ^i^	27.018 ^cd^
	IR 36	51.361 ^hi^	28.107 ^d^
	Kasalath IC459373	50.195 ^i^	30.659 ^a^
High P	CR Dhan 201	51.944 ^gh^	23.874 ^fg^
	CR Dhan 204	46.147 ^kL^	21.448 ^h^
	CR Dhan 205	47.297 ^jk^	23.452 ^fg^
	CR Dhan 207	45.574 ^L^	21.679 ^h^
	IR 36	50.774 ^hi^	24.290 ^ef^
	Kasalath IC459373	47.872 ^j^	22.583 ^gh^
CD(0.05)		1.623	1.457

Lowercase letters represent significant variations among the data at *p* < 0.05. Values represent the means of three replicates. Values (AMF sporulation) in parentheses are arsine-transformed values. Means followed by a common letter are not significantly different at 5% level by DMRT.

## Data Availability

Data are contained within the article and Supplementary Materials.

## Supplementary Materials

The following supporting information can be downloaded at: https://www.mdpi.com/article/10.3390/plants15111675/s1, Supplementary Table S1. Quantification of RNA concentration of all rice variety samples grown under low, medium, and high available soil P conditions with AMF inoculation. Supplementary Table S2. Quantification of RNA concentration of all rice variety samples grown under low, medium, and high available soil P conditions without AMF inoculation. Supplementary Table S3. Details and sequences of primers of *OsPT1*–*OsPT13* genes used in this study. Supplementary Figure S1. Low phosphorus soil sampling site. Supplementary Figure S2. Phosphorus nutrients (low, medium, and high) in experimental soil are altered by using K_2_HPO_4_. Supplementary Figure S3. AMF multiplication using the trap culture method. Supplementary Figure S4.1. AMF colonization in different rice varieties (CR Dhan 201, 204, 205, 207, Kasalath IC459373, and IR36) under low-P conditions. Supplementary Figure S4.2. AMF colonization in different rice varieties (CR Dhan 201, 204, 205, 207, Kasalath IC459373, and IR36) under medium-P conditions. Supplementary Figure S4.3. AMF colonization in different rice varieties (CR Dhan 201, 204, 205, 207, Kasalath IC459373, and IR36) under high-P conditions. Supplementary Figure S5. Chlorophyll fluorescence imaging. [AM1: with AM fungi; AM2: without AM fungi; Fo- minimum fluorescence when all the reaction centers are open; Fm- maximum fluorescence when all the reaction centers are closed; Fv- variable fluorescence (Fm − Fo); Fv/Fm = (1 − Fo/Fm); P1: Low P, P2: Medium P, P3: High P, V1: CR Dhan 201, V2: CR Dhan 204, V3: CR Dhan 205, V4: CR Dhan 207, V5: IR 36 (P susceptible), V6: Kasalath IC459373 (P tolerant)] Supplementary Figure S6.1. Collection of root samples for scanning from different rice varieties grown under different P conditions. Supplementary Figure S6.2 Root scanning image in different rice varieties under low soil available P conditions. Supplementary Figure S6.3. Root scanning image in different rice varieties under medium soil available P conditions. Supplementary Figure S6.4. Root scanning image in different rice varieties under high soil available P conditions.
