# Supplementary material for: Arbuscular Mycorrhizal Fungi Modulate Variety-Specific Phosphate Transporter Gene Expression in Aerobic Rice Under Phosphorus-Limited Soil Conditions
Source: Plants (Basel). 2026 May 29;15(11):1675. doi: 10.3390/plants15111675 (PMC13258995; doi:10.3390/plants15111675)
Supplement: Supplementary file 1 [file plants-15-01675-s001.zip › Supplementary Figure.pdf]

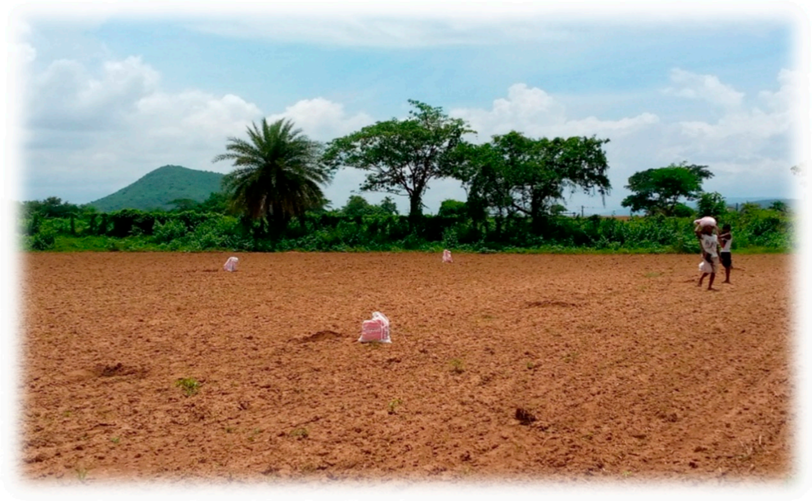

Supplementary Figure 1. Low phosphorus soil sampling site.

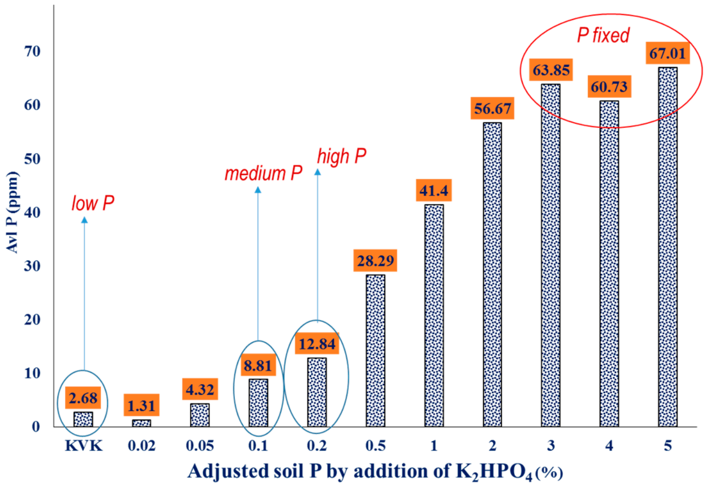

Supplementary Figure 2. Phosphorus nutrient contains (low, medium and high) in experimental soil alter by using K_2HPO_4.

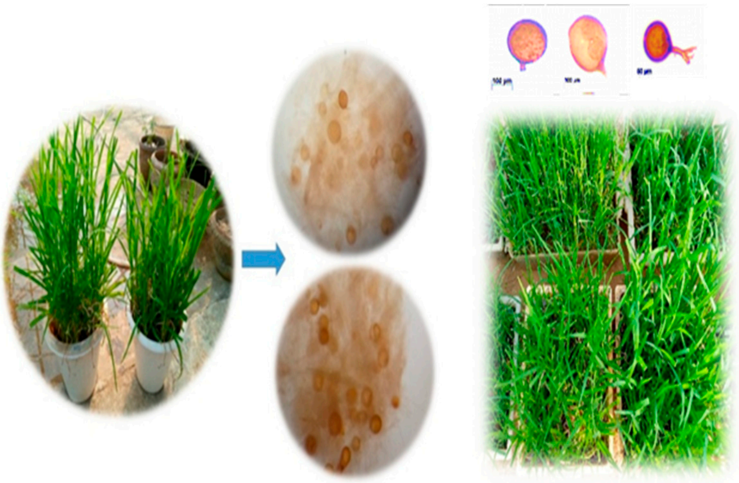

Supplementary Figure 3. AMF multiplication using trap culture method.

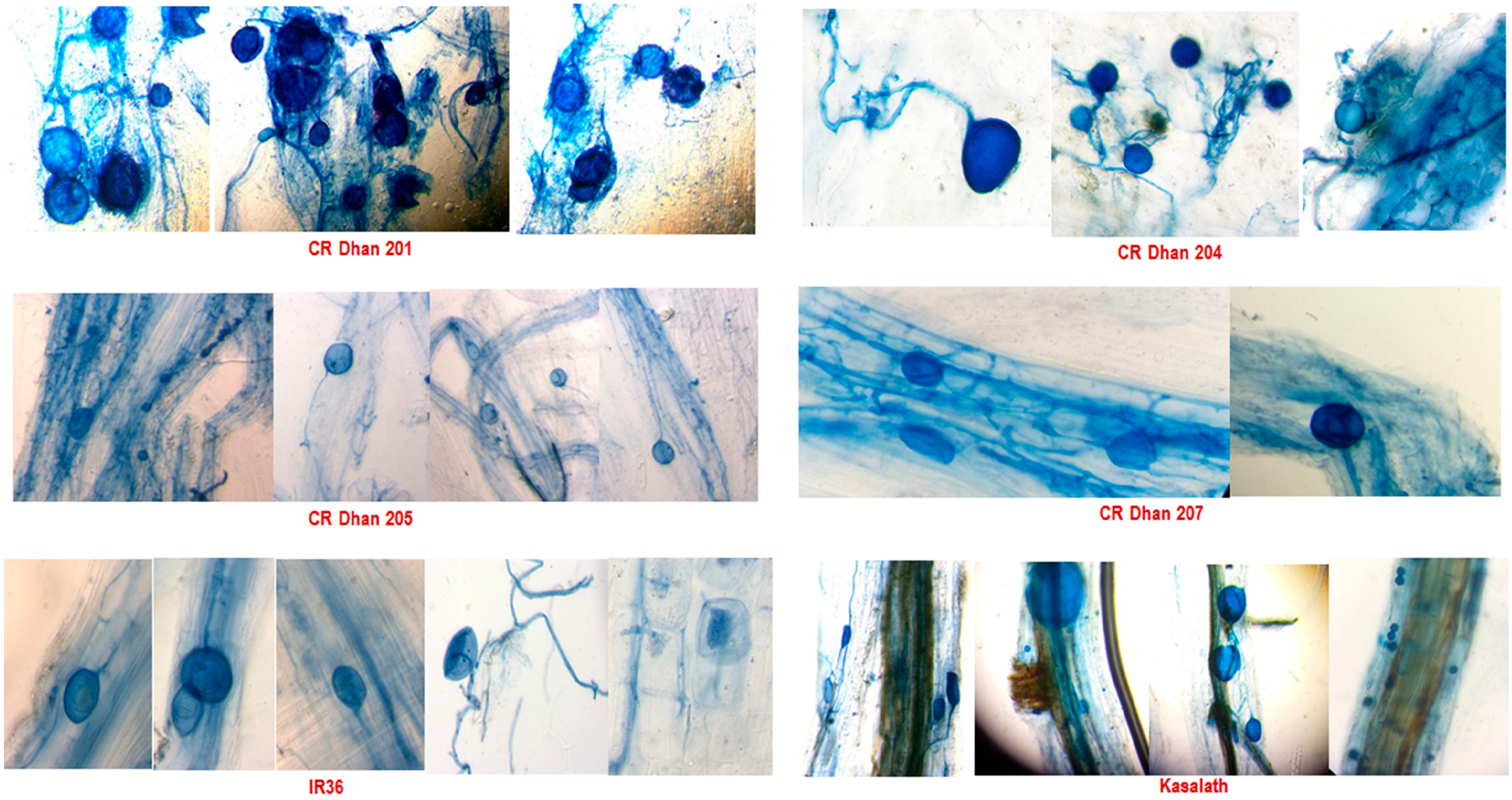

Supplementary Figure 4.1. AMF colonization in different rice varieties (CR Dhan 201, 204, 205, 207, Kasalath IC459373 and IR36) **under low P conditions.**

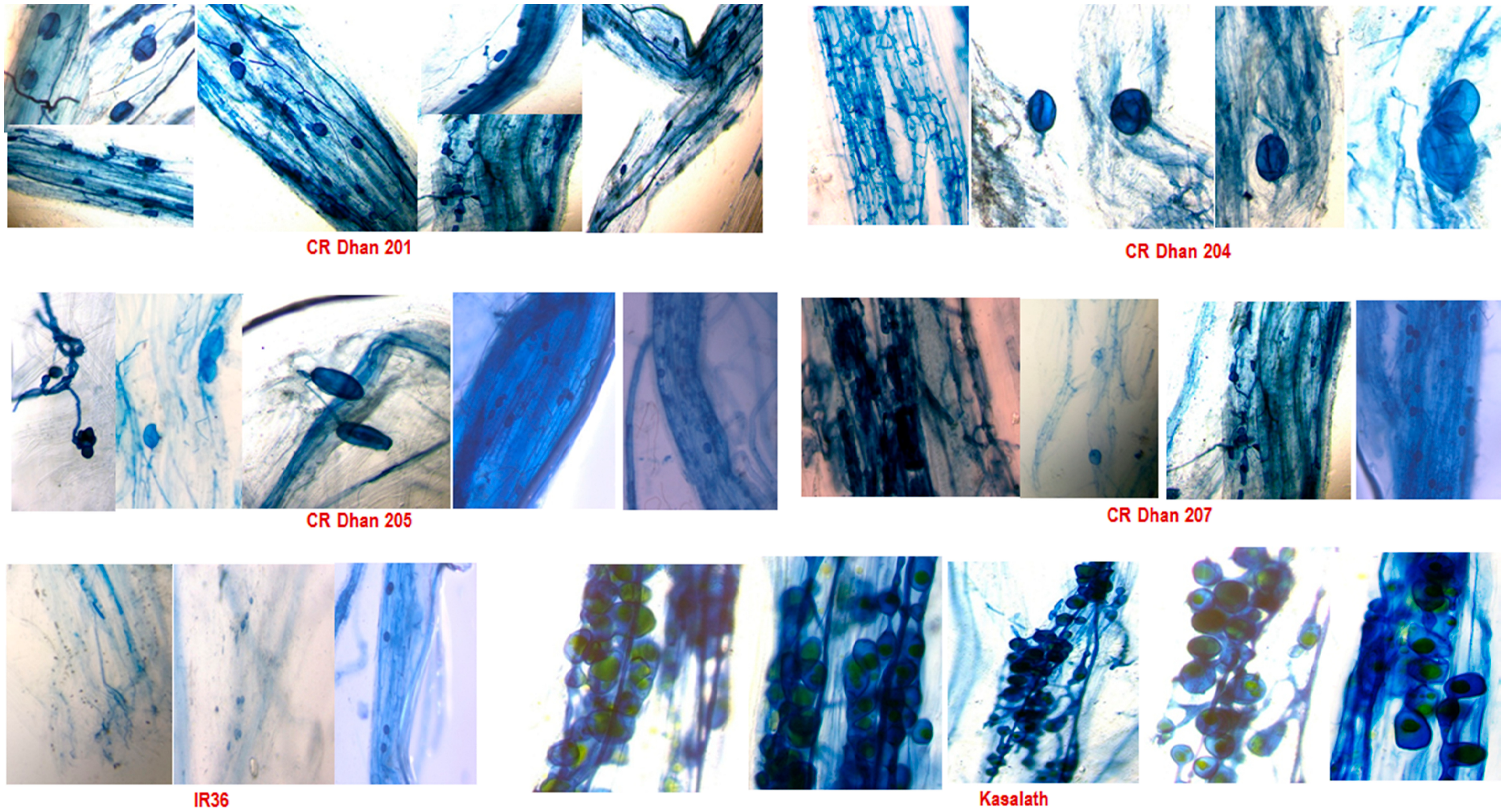

Supplementary Figure 4.2. AMF colonization in different rice varieties (CR Dhan 201, 204, 205, 207, Kasalath IC459373 and IR36) **under medium P conditions.**

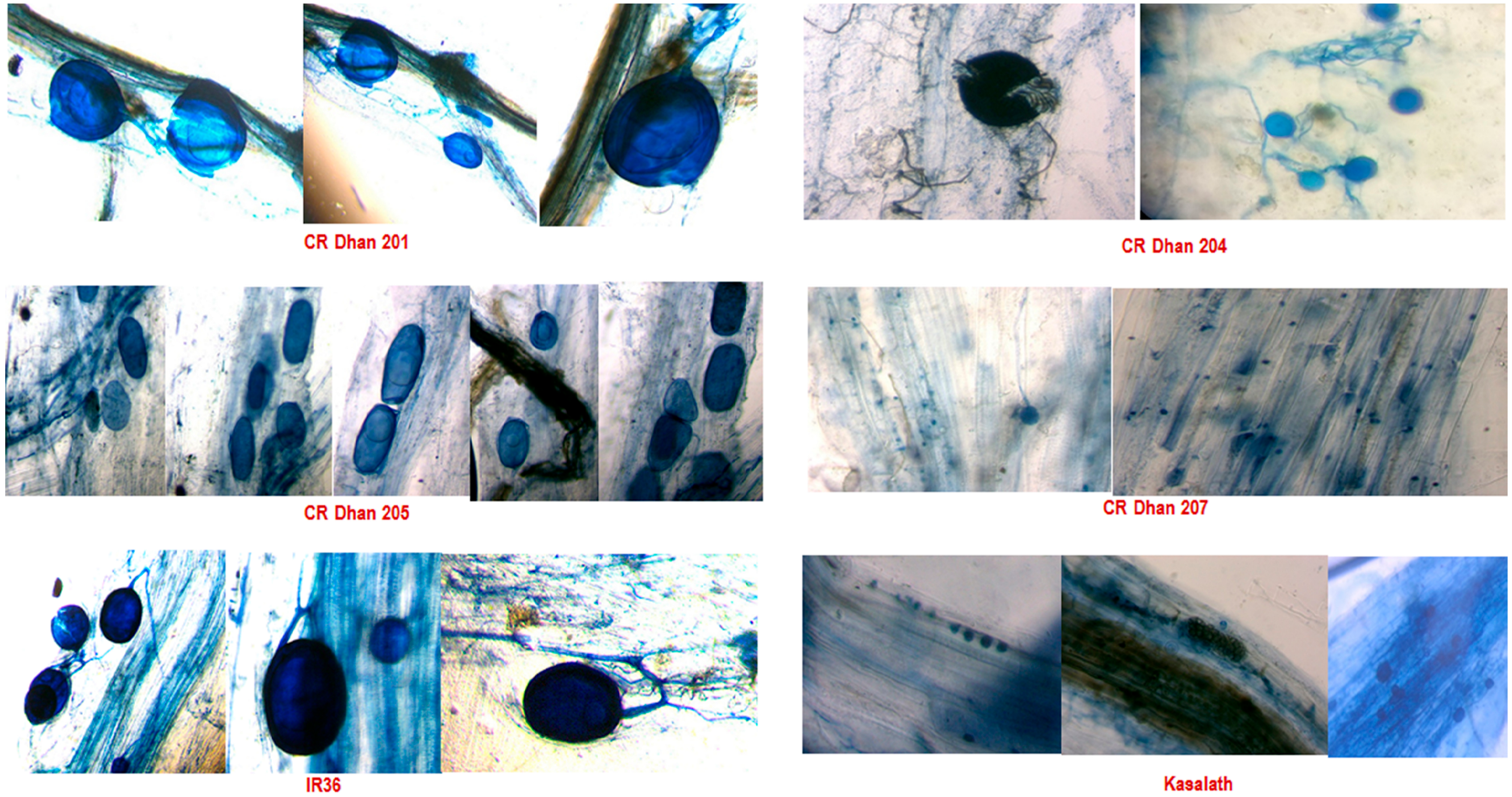

Supplementary Figure 4.3. AMF colonization in different rice varieties (CR Dhan 201, 204, 205, 207, Kasalath IC459373 and IR36) **under high P conditions.**

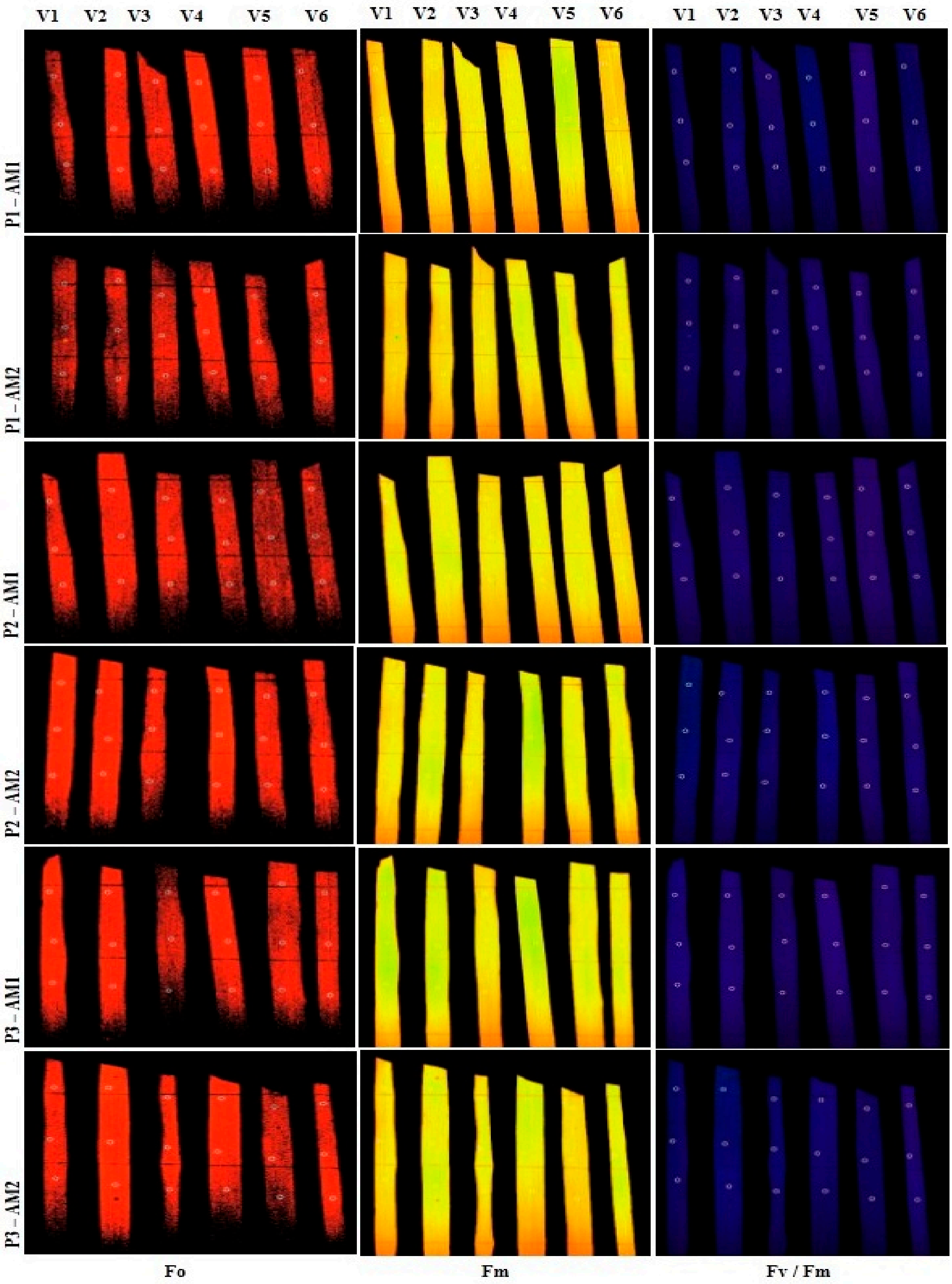

Supplementary Figure 5. Chlorophyll fluorescence imaging. [AM1: with AM fungi; AM2: without AM fungi; F_o- minimum fluorescence when all the reaction centres are open; F_m- maximum fluorescence when all the reaction centres are closed; F_v- variable fluorescence (F_m–F_o); $F_v/F_m=(1–F_o/F_m)$; P1: Low P, P2: Medium P, P3: High P, V1: CR Dhan 201, V2: CR Dhan 204, V3: CR Dhan 205, V4: CR Dhan 207, V5: IR 36 (P susceptible), V6: Kasalath IC459373 (P tolerant)]

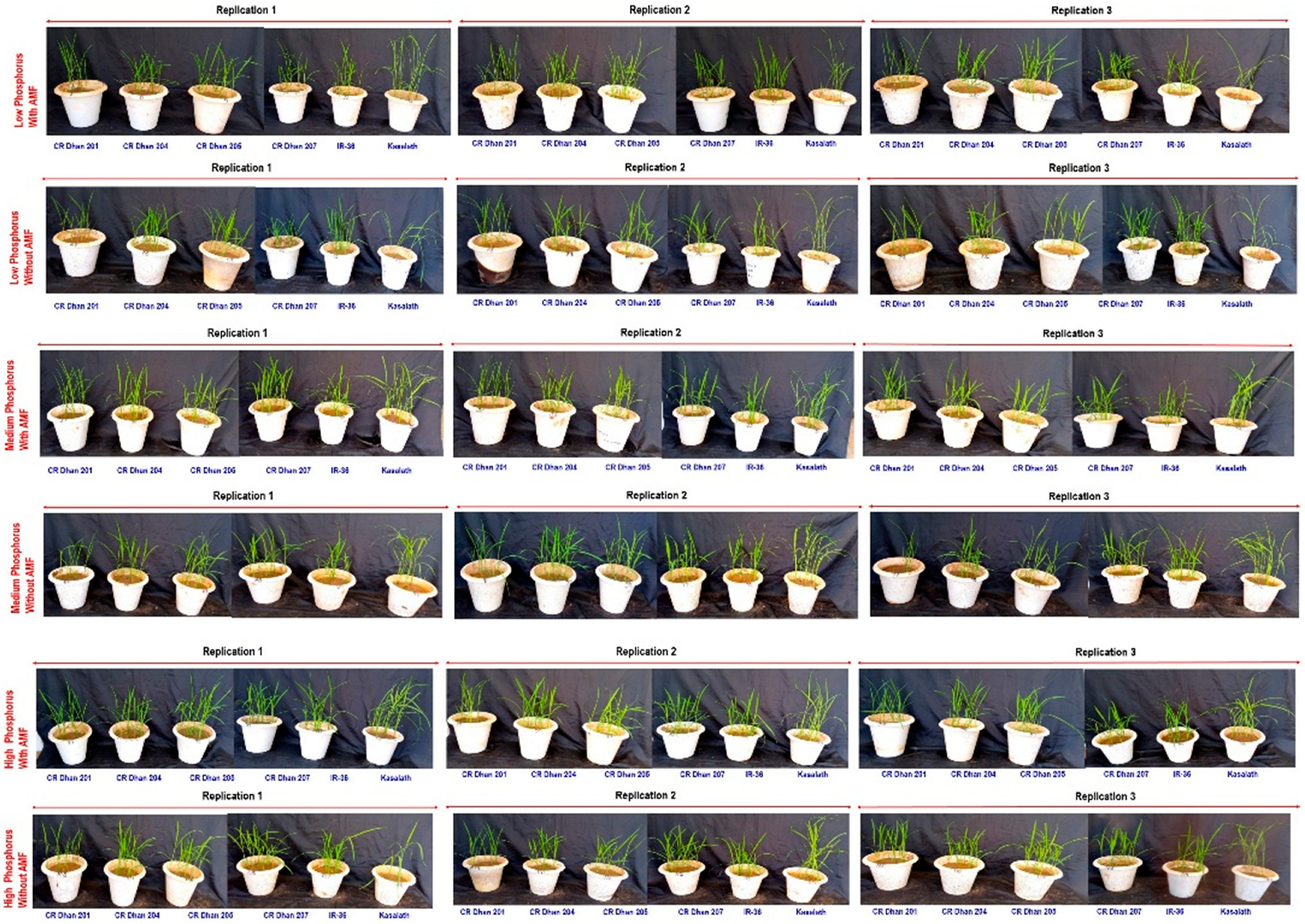

Supplementary Figure 6.1. Collection of root samples for scanning from different rice varieties grown under different P

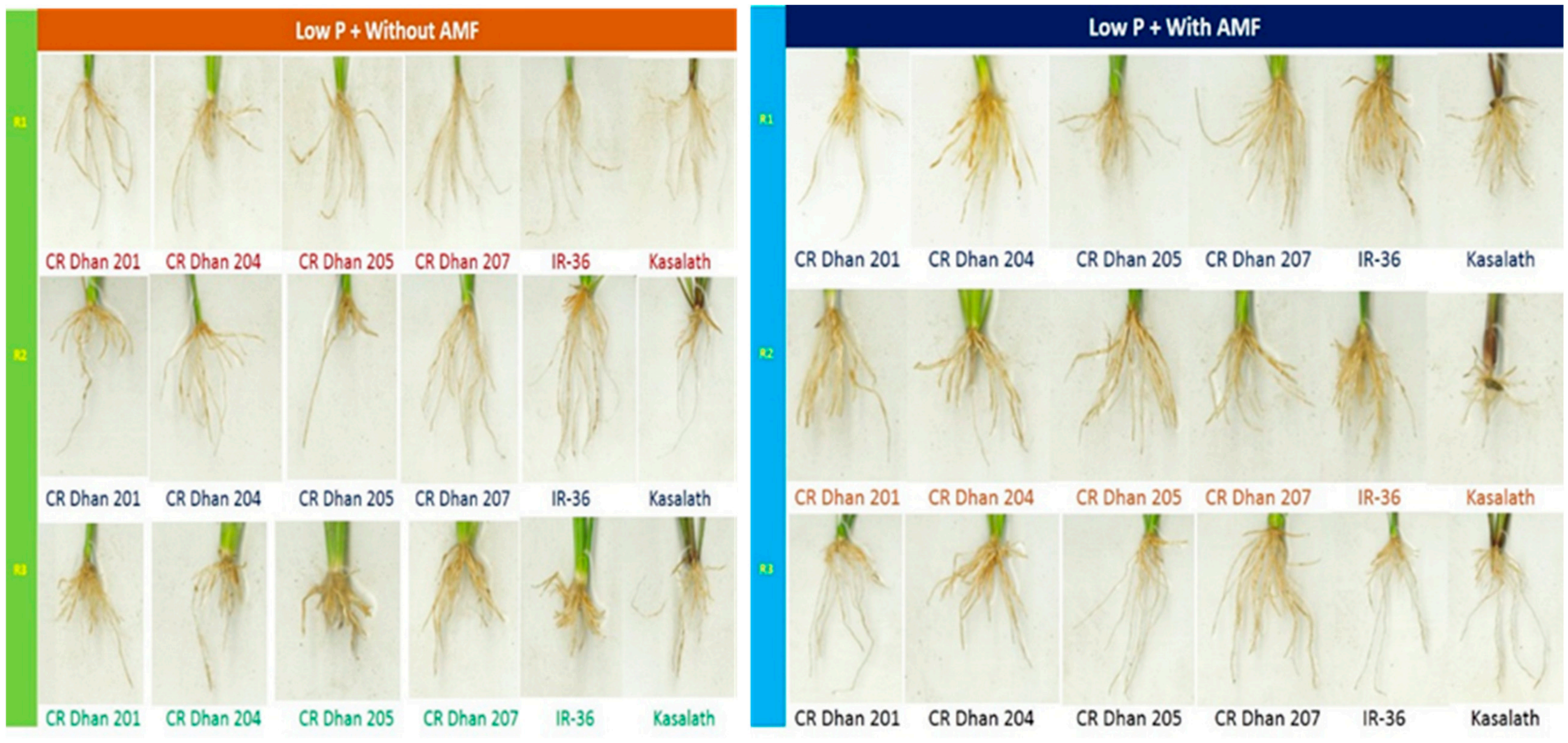

Supplementary figure 6.2 Root scanning image in different rice varieties **under low soil available P condition.**

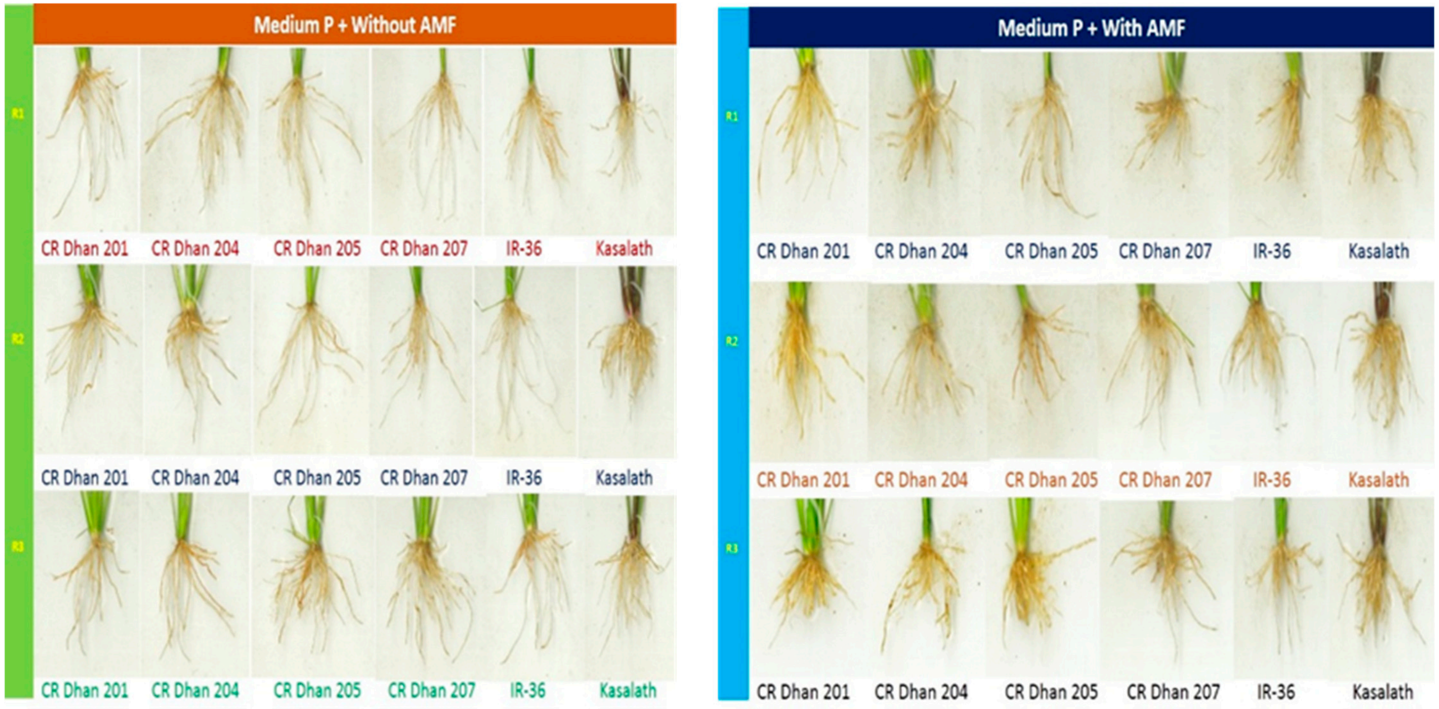

Supplementary figure 6.3. Root scanning image in different rice varieties **under medium soil available P condition.**

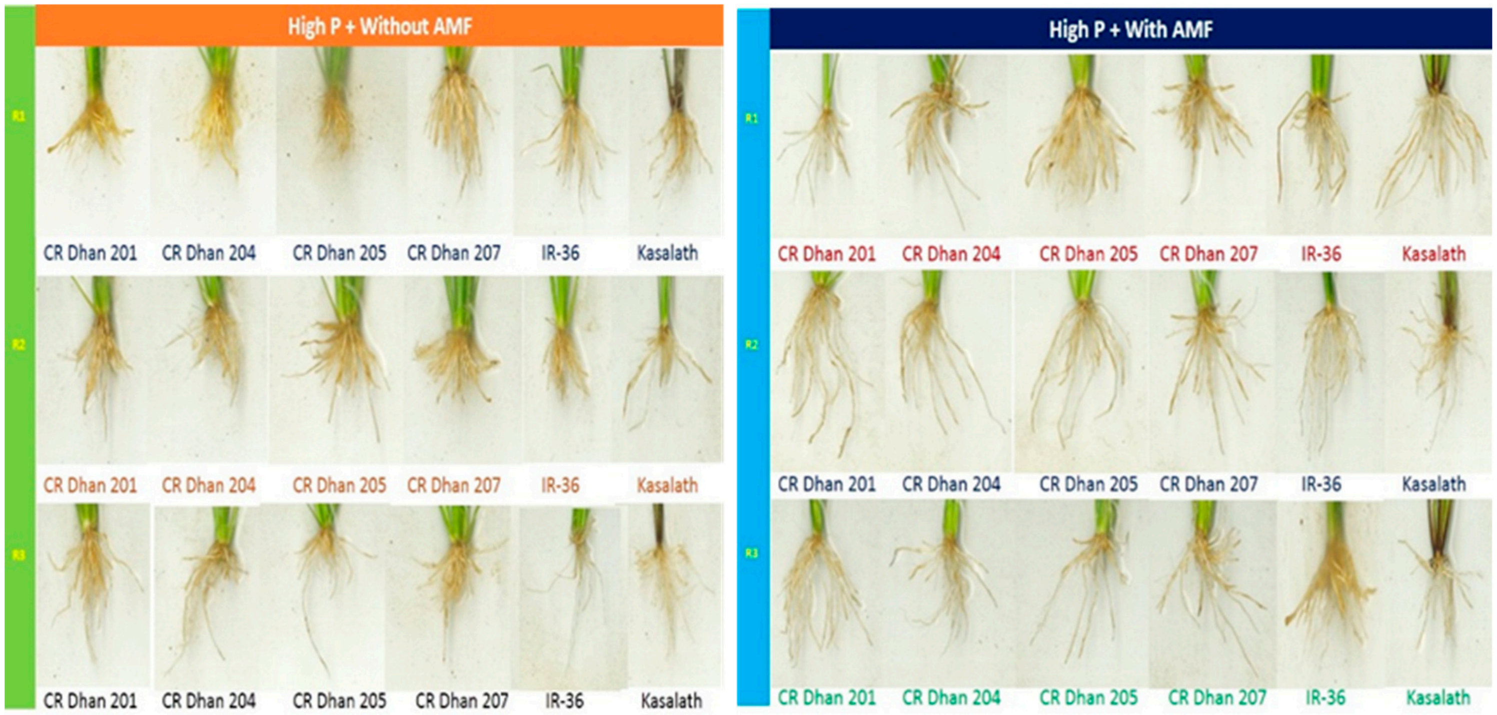

Supplementary figure 6.4. Root scanning image in different rice varieties **under high soil available P condition.**
