# Supplementary material for: Arbuscular Mycorrhizal Fungi Modulate Variety-Specific Phosphate Transporter Gene Expression in Aerobic Rice Under Phosphorus-Limited Soil Conditions
Source: Plants (Basel). 2026 May 29;15(11):1675. doi: 10.3390/plants15111675 (PMC13258995; doi:10.3390/plants15111675)
Supplement: Supplementary file 1 [file plants-15-01675-s001.zip › Supplementary Table.pdf]

Supplementary Table S1. Quantification of RNA concentration of all rice variety samples grown under low, medium and high available soil P condition **with AMF inoculation**.

	Varieties	RNA concentration (ng/µl)	$A_{260/280}$	$A_{260/230}$
Low P	**CR Dhan 201**	421.20 ± 7.03	1.92 ± 0.09	1.97 ± 0.56
	**CR Dhan 204**	130.30 ± 2.97	2.03 ± 0.19	2.07 ± 0.02
	**CR Dhan 205**	179.00 ± 2.63	2.01 ± 0.11	1.62 ± 0.48
	**CR Dhan 207**	207.20 ± 1.33	1.99 ± 0.08	1.68 ± 0.63
	**IR36**	264.80 ± 5.33	2.00 ± 0.19	1.89 ± 0.92
	**Kasalath IC459373**	212.40 ± 3.44	1.99 ± 0.22	1.53 ± 0.93
Medium P	**CR Dhan 201**	165.70 ± 6.33	2.01 ± 0.12	1.66 ± 0.93
	**CR Dhan 204**	201.90 ± 4.43	1.94 ± 0.36	1.59 ± 0.19
	**CR Dhan 205**	225.5 ± 5.34	2.00 ± 0.31	1.55 ± 0.75
	**CR Dhan 207**	277.90 ± 3.34	1.95 ± 0.11	1.73 ± 0.36
	**IR36**	161.30 ± 2.09	2.00 ±0.09	1.67 ± 0.54
	**Kasalath IC459373**	133.90 ± 2.44	2.03 ± 0.21	1.46 ± 0.47
High P	**CR Dhan 201**	281.50 ± 5.54	2.00 ± 0.32	1.46 ± 0.37
	**CR Dhan 204**	226.00 ± 8.44	2.00 ± 0.13	1.76 ± 0.83
	**CR Dhan 205**	298.90 ± 3.09	1.99 ± 0.19	1.79 ± 0.24
	**CR Dhan 207**	139.30 ± 9.37	1.94 ± 0.22	0.80 ± 0.75
	**IR36**	336.50 ± 6.44	1.93 ± 0.31	1.49 ± 0.46
	**Kasalath IC459373**	254.80 ± 5.33	2.01 ± 0.19	1.81 ± 0.84

*Standard deviation (S.D.) is represented as ±, which was calculated from the replicated values.

Supplementary Table S2. Quantification of RNA concentration of all rice variety samples grown under low, medium and high available soil P condition **without AMF inoculation.**

	Varieties	RNA concentration (ng/µl)	$A_{260/280}$	$A_{260/230}$
Low P	CR Dhan 201	808.07 ± 3.20	2.09 ± 0.05	1.98 ± 0.27
	CR Dhan 204	119.10 ± 2.36	2.03 ± 0.03	1.10 ± 0.43
	CR Dhan 205	265.70 ± 1.38	2.04 ± 0.10	1.73 ± 0.19
	CR Dhan 207	479.70 ± 4.48	2.05 ± 0.09	2.17 ± 0.09
	IR36	309.40 ± 9.98	2.08 ± 0.12	2.22 ± 0.11
	Kasalath IC459373	373.00 ± 4.38	2.09 ± 0.05	1.14 ± 0.56
Medium P	CR Dhan 201	1320.00 ± 2.38	2.08 ± 0.09	2.22 ± 0.13
	CR Dhan 204	260.03 ± 8.37	2.02 ± 0.13	1.16 ± 0.45
	CR Dhan 205	504.20 ± 9.47	2.05 ± 0.24	2.15 ± 0.86
	CR Dhan 207	235.20 ± 2.47	2.05 ± 0.04	1.91 ± 0.19
	IR36	460.10 ± 4.38	2.08 ± 0.35	1.80 ± 0.12
	Kasalath IC459373	315.10 ± 2.47	2.05 ± 0.13	2.12 ± 0.08
High P	CR Dhan 201	309.90 ± 6.47	2.10 ± 0.09	1.06 ± 0.32
	CR Dhan 204	686.20 ± 3.48	2.07 ± 0.12	2.33 ± 0.14
	CR Dhan 205	392.00 ± 6.47	2.09 ± 0.23	1.31 ± 0.56
	CR Dhan 207	482.50 ± 2.48	2.10 ± 0.10	1.52 ± 0.48
	IR36	589.70 ± 5.73	2.09 ± 0.08	2.37 ± 0.08
	Kasalath IC459373	385.10 ± 3.47	2.08 ± 0.11	2.27 ± 0.10

*Standard deviation (S.D.) is represented as ±, which was calculated from the replicated values.

Supplementary Table S3. Details and sequences of primers of *OsPT1-OsPT13* genes used in this study.

Primer name	Sequence (5´ - 3´)
OsPT1-F	CGCTTCCGTACGAGTGGTAGT
OsPT1-R	GGTTCTTTCAAATCCAGGGAAA
OsPT2-F	AGCTGTTGGGTCGCCTTTACTACA
OsPT2-R	ACGACCATGAGGATGAGCGTGAAT
OsPT3-F	TGCGACTGCTGATTCAGTACGT
OsPT3-R	ACAAATGCCATCAAATATGAACAGA
OsPT4-F	GGTTCACCGTCTTCTTCATCGACA
OsPT4-R	AGAAGGTGAAGGCGTACATGACCA
OsPT5-F	TGCTACTGCCCATGACTAGGATT
OsPT5-R	CCATAGAAGAGATCCAGAGAAGCTGTA
OsPT6-F	ACGCTCTCAGGGCAACTCTTCTTT
OsPT6-R	TGAGCATGAGGGGTATGCCATAGA
OsPT7-F	TCCAAGGTGCTCCAGGTGAAGAT
OsPT7-R	TCTGGAACAGGTTCTGGGAGTAGT
OsPT8-F	TCCAGAAGGACATCTTCACCAGCA
OsPT8-R	ATGTCGATGAGGAAGACGGTGAAC
OsPT9-F	AGAAAAACATAGGCTTGTCATCCTTT
OsPT9-R	AAAACCTAAGAAGCACTGTAAATAAAT
OsPT10-F	ATGTCGCCCATCCTTCCA
OsPT10-R	TCGCTTTCCGACGATGATC
OsPT11-F	AAGTTCAACGCGGCCAACAACTAC
OsPT11-R	AACATCTCCGTGAGAGCGTTGACT
OsPT12-F	TCATCGGAGCATTCGGTTTCCTCT
OsPT12-R	ATGTCATGAGGAACCCAACGAGGT
OsPT13-F	GGTGCCTTCATTGCTGCTGTCTTT
OsPT13-R	TGCTGTTTCTGGCATCTTCATGCG

F – forward primer; R – reverse primer.
